# Regulation of DNA‐PK activity promotes the progression of TNBC via enhancing the immunosuppressive function of myeloid‐derived suppressor cells

**DOI:** 10.1002/cam4.5387

**Published:** 2022-11-13

**Authors:** Jiawen Han, Minjie Wan, Zhanchuan Ma, Huanfa Yi

**Affiliations:** ^1^ Central Laboratory The First Hospital of Jilin University Changchun China; ^2^ Key Laboratory of Organ Regeneration and Transplantation Ministry of Education Changchun China; ^3^ Department of Hepatology The First Hospital of Jilin University Changchun China

**Keywords:** DNA‐PK, immunosuppressive function, MDSCs, NU7441, TNBC, tumor progression

## Abstract

**Background:**

DNA‐dependent protein kinase (DNA‐PK) is engaged in DNA damage repair and is significantly expressed in triple negative breast cancer (TNBC). Inhibiting DNA‐PK to reduce DNA damage repair provides a possibility of tumor treatment. NU7441, a DNA‐PK inhibitor, can regulate the function and differentiation of CD4^+^T cells and effectively enhance immunogenicity of monocyte‐derived dendritic cells. However, the effect of NU7441 on the tumor progression activity of immunosuppressive myeloid‐derived suppressor cells (MDSCs) in TNBC remains unclear.

**Results:**

In this study, we found that NU7441 alone significantly increased tumor growth in 4 T1 (a mouse TNBC cell line) tumor‐bearing mice. Bioinformatics analysis showed that DNA‐PK and functional markers of MDSCs (*iNOS*, *Arg1*, and *IDO*) tended to coexist in breast cancer patients. The mutations of these genes were significantly correlated with lower survival in breast cancer patients. Moreover, NU7441 significantly decreased the percentage of MDSCs in peripheral blood mononuclear cells (PBMCs), spleen and tumor, but enhanced the immunosuppressive function of splenic MDSCs. Furthermore, NU7441 increased MDSCs' DNA‐PK and pDNA‐PK protein levels in PBMCs and in the spleen and increased DNA‐PK mRNA expression and expression of MDSCs functional markers in splenic MDSCs from tumor‐bearing mice. NU7441 combined with gemcitabine reduced tumor volume, which may be because gemcitabine eliminated the remaining MDSCs with enhanced immunosuppressive ability.

**Conclusions:**

These findings highlight that the regulation of DNA‐PK activity by NU7441 promotes TNBC progression via enhancing the immunosuppressive function of MDSCs. Moreover, NU7441 combined with gemcitabine offers an efficient therapeutic approach for TNBC and merits deeper investigation.

## Graphical abstract

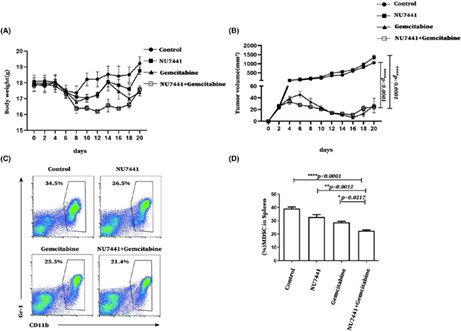

## 1 INTRODUCTION

Breast cancer is one of the most prevalent cancers affecting women's health.^1^ Despite a significant improvement in overall survival, it remains a major factor of death all around the world.^1^ Based on the expression of estrogen or progesterone receptors and human epidermal growth factor 2 (ERBB2; previously known as HER2) molecular markers, the breast cancer subtypes can be categorized into three groups: hormone‐receptor positive/ERBB2 negative (70%), ERBB2 positive (15%–20%), and triple negative (15%).^2^ Triple‐negative breast cancer (TNBC) is the deadliest breast cancer subtype owing to high heterogeneity, aggressiveness, and shortage of treatment options.^3^ Despite many years of research, it is still necessary to explore how to better manage TNBC.

Solid tumor progression is affected by tumor microenvironment, which suppresses anti‐tumor immune response and supports disease progression.^4^ Myeloid‐derived suppressor cells (MDSCs) are a cluster of heterogeneous immature myeloid cells that consist of immature monocytes, neutrophils and dendritic cells (DCs).^5^ MDSCs are important contributors to the immunosuppressive ability of the tumor microenvironment, show effective immunosuppressive ability, and are present in most cancer types.^6^ Generally, MDSCs can be classified into two subgroups based on different markers, namely polymorphonuclear (PMN‐MDSCs) and monocyte (Mo‐MDSC) types, which are defined by their surface phenotypes and functions.^7^ In mice, MDSCs are poorly differentiated and heterogeneous CD11b + Gr1+ cells, which can be further classified into two subgroups which include polymorphonuclear (PMN‐MDSCs; Ly6G+) and mononuclear (Mo‐MDSCs; Ly6C+)^8^ In humans, PMN‐MDSCs are defined by immature medullary markers such as CD14‐CD11b + CD33 + CD15 + LOX‐1+ and CD66B+ cells, while Mo‐MDSCs include CD14+ and HLA‐DR−/low cells.^8^ They have an outstanding capacity to suppress T‐cell responses through producing reactive oxygen species (ROS), arginase‐1 (Arg‐1), and nitric oxide (NO).^9^ Trauma, infection, malignancy, and aberrant inflammatory states trigger MDSC proliferation, then MDSCs are recruited to the inflammatory microenvironment, exerting immunosuppressive effects via secreting Arg‐1, inducible nitric oxide synthase (iNOS) and indolamine dioxygenase (IDO), which restrict TCR ζ‐chain production and prompt T‐cell apoptosis.^10^ The primary function of MDSCs is immunosuppression, which occurs through various mechanisms, including inducing immunosuppressive cells, impairing lymphocyte homing, producing free radical, depleting amino acids crucial for T cell functions, upregulating ectoenzymes included in adenosine production and activating immune regulatory molecules accountable for T cell anergy.^11^ The levels of MDSCs differ among patients with various forms of malignancies, and an increase in MDSCs was found in patients with TNBC.^12^ In addition, some studies have shown that TNBC can be treated by regulating MDSCs in different ways. Firstly, eliminating MDSCs an effectively inhibit the progression and metastasis of TNBC. One study found that eliminating MDSCs through gemcitabine administration significantly inhibited tumor growth and reduced splenomegaly.^13^ Also, eliminating MDSCs by DABIL‐4 (a fusion protein toxin comprising the catalytic and translocation domains of diphtheria toxin fused to murine IL‐4) can also significantly reduce tumor growth, splenomegaly, and lung metastases.^14^ Secondly, preventing the accumulation and recruitment of MDSCs is also considered an effective treatment strategy for TNBC. According to prior studies, preventing the accumulation and recruitment of MDSCs by CXCR2/CCR4 inhibitors reduced angiogenesis and metastasis.^12^ Besides, sulforaphane combined with doxorubicin can prevent MDSCs accumulation to further suppress breast cancer growth.^15^ Preventing MDSCs accumulation and recruitment by a clinical‐stage monoclonal IL‐8 neutralizing antibody (HuMax‐IL8) can also reduce tumor growth.^16^ Lastly, regulating metabolism is also regarded as a new treatment direction for TNBC. A prior study has shown that activated MEK can modulate glycolysis and support MDSCs in TNBC, thus promoting tumor progression.^17^ Besides, tumor glycolysis can regulate AMPK‐ULK1, autophagy, and CEBPB pathways to affect MDSCs and suppress anti‐tumor immune response in TNBC.^18^ Therefore, MDSCs play a significant part in TNBC progression. Regulation of MDSCs is a potential future direction for TNBC treatment.

DNA‐dependent protein kinase (DNA‐PK) is a pleiotropic kinase involved in DNA repair and transcriptional regulation, which play roles in a variety of biological processes.^19^, ^20^ As a key enzyme in the non‐homologous end joining (NHEJ) pathway of double strand break (DSB), DNA‐PK is significantly expressed in breast cancer including TNBC and certain cancer types (non‐small cell lung cancer, prostate cancer, and liver cancer), and is intimately connected to the dreadful prognosis of breast cancer.^21^, ^22^ DNA‐PK has been previously shown to modulate cancer phenotypes through DNA repair via NHEJ and transcriptional regulatory mechanisms.^23^ In addition, DNA‐PK also mediates epithelial‐mesenchymal transition, tumor metastasis and metabolic processes in cancer. Previous studies have described DNA‐PK to directly interact with the EMT protein Snail and drive metastasis.^24^ Previously published data have also linked DNA‐PK to play a pro‐metastatic role in cancer.^23^ Another study in melanoma has shown that DNA‐PK modifies the tumor microenvironment by modulating the secretion of pro‐migratory molecules and promotes metastasis.^25^ Besides, DNA‐PK regulates cancer cell metabolism, including: fatty acid metabolism, cholesterol homeostasis, and oxidative phosphorylation.^26^ Overall, these findings suggest that DNA‐PK might be a potential therapeutic target for breast cancer, including TNBC. Reduction in the degree of repair of DSB through DNA‐PK inhibition offers the possibility of tumor therapy. DNA‐PK inhibitors were often used as radiotherapy and chemotherapy sensitizers in preclinical trials and usually combined with radiotherapy and chemotherapy since they are ineffective when used alone^27^, ^28^; the reason for this is not clear. Studies have shown that DNA‐PK inhibitor NU7441 can regulate the function and differentiation of CD4^+^ T cells^29^ and effectively enhance the immunogenicity of monocyte‐derived dendritic cells (moDC).^30^ Nevertheless, no studies have been done on how NU7441 affects MDSCs yet. This research was dedicated to evaluate the effects of NU7441 on MDSCs in vivo and in vitro, its mechanism, and the effect of NU7441 combined with gemcitabine on TNBC tumor growth.

## 2 MATERIALS AND METHODS

### 2.1 Gene expression and survival analysis

Gene expression and survival analysis were obtained from cBioPortal web server (http://cbioportal.org), which is an open web that contains 9736 tumors of 33 cancer types and 8587 normal samples from TCGA and GTEx projects.^31^ cBioPortal, an online tool that allows users to explore and visualize multidimensional cancer genome datasets,^32^ was used to predict the coexistence tendency of DNA‐PK and common MDSC function‐related genes (NOS2, ARG1, and IDO1) and visualize the survival time of patients with breast cancer with gene mutation.

### 2.2 Cell line

The TNBC cell line 4 T1 was purchased from the Shanghai Institute of Cell Biology, Chinese Academy of Sciences (Shanghai, China) and cultured in DMEM medium supplemented with 10% fetal bovine serum (FBS) (Gibco). They were cultured at 37°C in a humidified 5% CO2 atmosphere. Mid‐log phase cells were applied for the following researches.

### 2.3 Animals

Female mice aged 6–8 weeks were bred in SPF animal room for animal experimentation. BALB/c mice were used to construct TNBC models. All the mice were from Viton Lever (Beijing, China). All animal experiments were approved by the Subcommittee on Research Animal Care of the First Hospital of Jilin University.

### 2.4 Animal model construction and drug treatment

1 × 10^6^ 4 T1 cells resuspended with PBS were injected into the mammary fat pads of BALB/c female mice in groups A, B, C, and D per mouse, to construct orthotopic breast cancer mouse model. NU7441 was purchased from TOPSCIENCE (Shanghai, China). Upon receipt, it was dissolved in dimethyl sulfoxide then stored in a 10 mM storage solution under sterile conditions at −80°C. 4 T1 cells and sorted MDSC cells were cultured in DMEM/RPMI 1640 medium containing 10% FBS. These cells were placed in 6‐well plates and treated with NU7441 (1 μM) for 16 h. In addition, from Day 7 after the injection of 4 T1 cells, NU7441 (dissolved in saline containing 40% PEG400) was administered intraperitoneally at a dose of 10 mg/kg once a day for 5 consecutive days to tumor‐bearing mice. At the same time, from day 7 after the injection of 4 T1 cells, the solvent (saline containing 40% PEG400) was administered intraperitoneally at the same volume once a day for 5 consecutive days to tumor‐bearing mice in the control group. Gemcitabine was purchased from HANSOH PHARMA (Lianyungang, China), sealed, and sterilized. Gemcitabine (dissolved in saline) was administered intraperitoneally at a dose of 100 mg/kg to tumor‐bearing mice on days 3, 6, 9, and 12 after the injection of 4 T1 cells. The tumors were measured every 3.5 days and their volumes were calculated as follows: volume = (length × width^2^)/2.

### 2.5 Cell isolation

Female BALB/c mice (6–8 weeks) were raised under certain pathogen‐free (SPF) conditions. Then, 4 T1 cells were cultured in DMEM supplemented with 10% FBS in 5% CO2 incubators. 4 T1 cells (1 × 10^6^ cells in 100 μl PBS) were injected into the mammary fat pads of BALB/c mice and treated with NU7441 for 5 consecutive days after Day 7. PBMCs were extracted and isolated prior to the development of the tumor, pre‐NU7441 administration, and after 5 consecutive days of NU7441 administration in the tumor‐bearing mice. Splenic cells and tumor infiltrating immune cells were isolated from tumor‐bearing mice when they were killed. These were then cultured with RPMI 1640 medium supplemented with 10% FBS.

### 2.6 Cell sorting

To sort MDSC from mouse, 1 × 10^6^ 4 T1 cells were injected into the mammary fat pads of BALB/c mice. The spleen from the tumor‐bearing mice was taken out 19 days later. Then, it was ground, split red, and filtered with a 70 μm filter to obtain single‐cell suspension. Anti‐mouse CD11b apccy7 (M1/70, Biolegend) and anti‐mouse Gr‐1 percpcy5.5 (RB6‐8C5, Biolegend) antibodies were used for staining splenic cell suspension at 4°C for 30 min. These were washed twice by cold PBS. CD11b^+^Gr1^+^ cells with purity higher than 95% were sorted from the splenic suspension and used in the subsequent research.

### 2.7 In vitro suppressive assays

The cells from the mouse lymph nodes were stained with 2.5 μM CFSE (eBioscience) for 15 min, then complete RPMI 1640 medium (HyClone) was added to stop the staining. After washing twice, the CFSE‐labeled cells were cultured in complete RPMI 1640 medium containing anti‐mouse CD28 purified antibody (1 μg/ml) for 72 h, in the 96‐well plate pre‐incubated with anti‐mouse CD3 purified antibody (2 μg/ml) at 4°C overnight.

MDSCs (CD11b^+^Gr‐1^+^ cells) were isolated from mice spleens. In short, the red blood cells were lysed first. Then, the splenic cells were washed with FACS buffer, and then incubated with CD11b apccy7 and Gr‐1 percpcy5.5 antibodies at 4°C for 30 min. MDSCs (CD11b^+^Gr‐1^+^ cells) (purity>95%) were then sorted and co‐cultured with T cells (MDSC: T = 1:2, MDSC: T = 1:1) for 72 h. Flow cytometry was applied to analyze T cell proliferation according to the dilution of CFSE.

### 2.8 Flow cytometry

For flow cytometry analysis, cells were collected, and the single‐cell suspension was washed twice by FACS buffer. Fluorescent‐labeled monoclonal antibodies were used to detect CD3^+^ T cells, Tregs, MDSCs, and the protein levels of DNA‐PK and pDNA‐PK in MDSCs. These antibodies are as follows: CD3 APC (17A2), CD4 PB (GK1.5), CD8 PE (53–6.7), CD11b apccy7 (M1/70), Gr‐1percpcy5.5 (RB6‐8C5), Ly 6C pecy7 (AL‐21), Ly 6G FITC (RB6‐8C5), Foxp3 percpcy5.5 (FJK‐16 s), DNA‐PK primary antibody (ab70250, Polyclonal), DNA‐PK‐pho primary antibody (phospho S2056) (ab18192, Polyclonal), secondary antibody goat anti‐rabbit IgG H&L Alexa Fluor® 405 (ab175652, Polyclonal), Fixable Viability Dye eFluor 506, and Fixable Viability Dye eFluor 780. Among these, Ly 6C and Ly 6G antibodies were purchased from BD Biosciences, and the remaining antibodies were purchased from Biolegend. The Ariall flow cytometer (BD Biosciences) was used for flow cytometry analysis while FlowJo software (version 7.6; FlowJo) was used for data evaluation.

### 2.9 Quantitative real‐time PCR

Using TRIzol reagent (Yeasen), total RNA was collected from 4 T1 cells and sorted splenic MDSCs. The extraction of gDNA and the synthesis of cDNA was carried out according to the manufacturer's instructions (Yeasen). StepOnePlus real‐time PCR system (Applied Biosystems) was used to run the qPCR using a commercial kit (Yeasen). The target gene mRNA levels were normalized to β‐actin. Moreover, the relative gene expression was calculated using the 2^−ΔΔCt^ method. The primer names and sequences are listed in Table S1.

### 2.10 Statistical analysis

Flow cytometry data were generated using FlowJo7.6 software, while data analysis was shown by Prism (version 7.0, GraphPad Software). T‐test was applied for comparing the differences between the two groups, one‐way ANOVA test was applied for comparing the differences between multiple groups, while two‐way ANOVA test was applied for comparisons involving two factor variables. All experiments were repeated three times, and all data were shown as mean ± SEM. *p* < 0.05 was regarded as significant.

## 3 RESULTS

### 3.1 NU7441 significantly promoted tumor growth in tumor‐bearing mice

**FIGURE 1:**
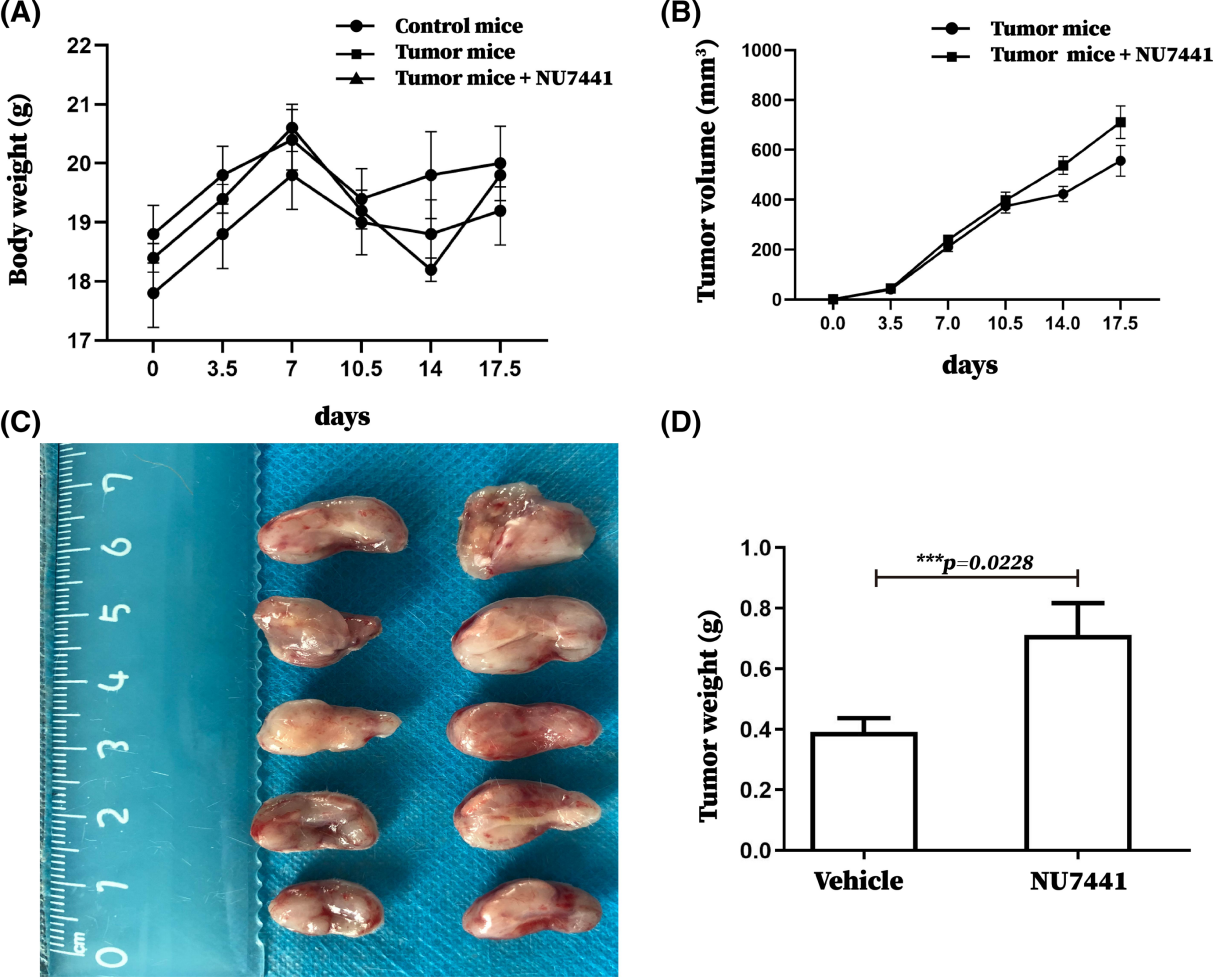
NU7441 significantly promoted tumor growth in tumor‐bearing mice. There were five mice in the control group. Tumor‐bearing mice were divided to 2 groups with 5 mice per group and intraperitoneally injected with DMSO or NU7441 at a dose of 10 mg/kg on the seventh day after tumor‐bearing, once a day for 5 consecutive days. (A) The body weight of the mice was recorded, the body weight curve of the mice was plotted from 3 independent experiments, (B) the tumor volume of the mice was recorded, the tumor volume curve of the mice was plotted from 3 independent experiments, (C) tumors were removed from mice at the time of death on the 18th day after tumor burden and were photographed, representative photo from 3 independent experiments was shown, (D) tumor weight from 3 independent experiments was recorded for data statistics. *p < 0.05; **p < 0.01; ***p < 0.001. The experiment was repeated three times, and the data were expressed in the form of (mean ± SEM).

We constructed a tumor‐bearing mice model and treated it with NU7441. NU7441 administration had no significant influence on mouse body weight (Figure 1A), indicating that NU7441 had no toxic side effects on mice. Additionally, NU7441 administration increased tumor volume and weight in tumor‐bearing mice (Figure 1B–D). Therefore, NU7441 significantly promoted tumor growth in tumor‐bearing mice. Subsequently, we explored the effect of NU7441 on DNA‐PK mRNA expression in 4 T1. However, DNA‐PK mRNA expression in 4 T1 cells after NU7441 administration was not of significant change (Figure S1). In addition, the effects of NU7441 on the protein levels of DNA‐PK and pDNA‐PK were examined by flow cytometry and results indicated that NU7441 administration significantly reduced pDNA‐PK levels without affecting DNA‐PK levels (Figure S2A,B), suggesting that NU7441 could inhibit DNA‐PK phosphorylation in 4 T1 cells. However, our results showed that NU7441 significantly promoted tumor growth in tumor‐bearing mice. According to these findings, the mechanism of NU7441 that is responsible in promoting tumor growth in tumor‐bearing mice was questioned.

### 3.2 DNA‐PK and MDSC immunosuppressive functions were correlated to lower overall survival in breast cancer patients

To investigate the specific mechanism of NU7441 promoting tumor growth in tumor‐bearing mice, we first conducted a study on the correlation between DNA‐PK and MDSC immunosuppressive function in breast cancer patients. Previously, no association between DNA‐PK and MDSCs had been reported. Using cBioPortal, it was found that *PRKDC* (DNA‐PK encoding gene) tended to coexist with the functional markers of MDSCs which include *NOS2* (iNOS‐encoding gene), *Arg1* (Arg1‐encoding gene), and *IDO1* (IDO‐encoding gene) in 19 breast cancer studies involving 9134 patients/9555 samples. This suggested that there may exist an interaction between DNA‐PK and MDSC immunosuppressive function in breast cancer patients and that regulating DNA‐PK may affect MDSC immunosuppressive function (Table 1).

**TABLE 1:** DNA‐PK and functional markers of MDSCs (*iNOS*, *Arg1*, and *IDO*) tend to coexist in breast cancer patients

A	B	Neither	A Not B	B Not A	Both	Log2 odds ratio	*p*‐value	q‐value	Tendency
*PRKDC*	*IDO1*	3860	219	288	83	2.345	<0.001	<0.001	Co‐occurrence
*PRKDC*	*NOS2*	3994	271	154	31	1.569	<0.001	<0.001	Co‐occurrence
*PRKDC*	*ARG1*	4092	288	56	14	1.829	<0.001	<0.001	Co‐occurrence

**FIGURE 2:**
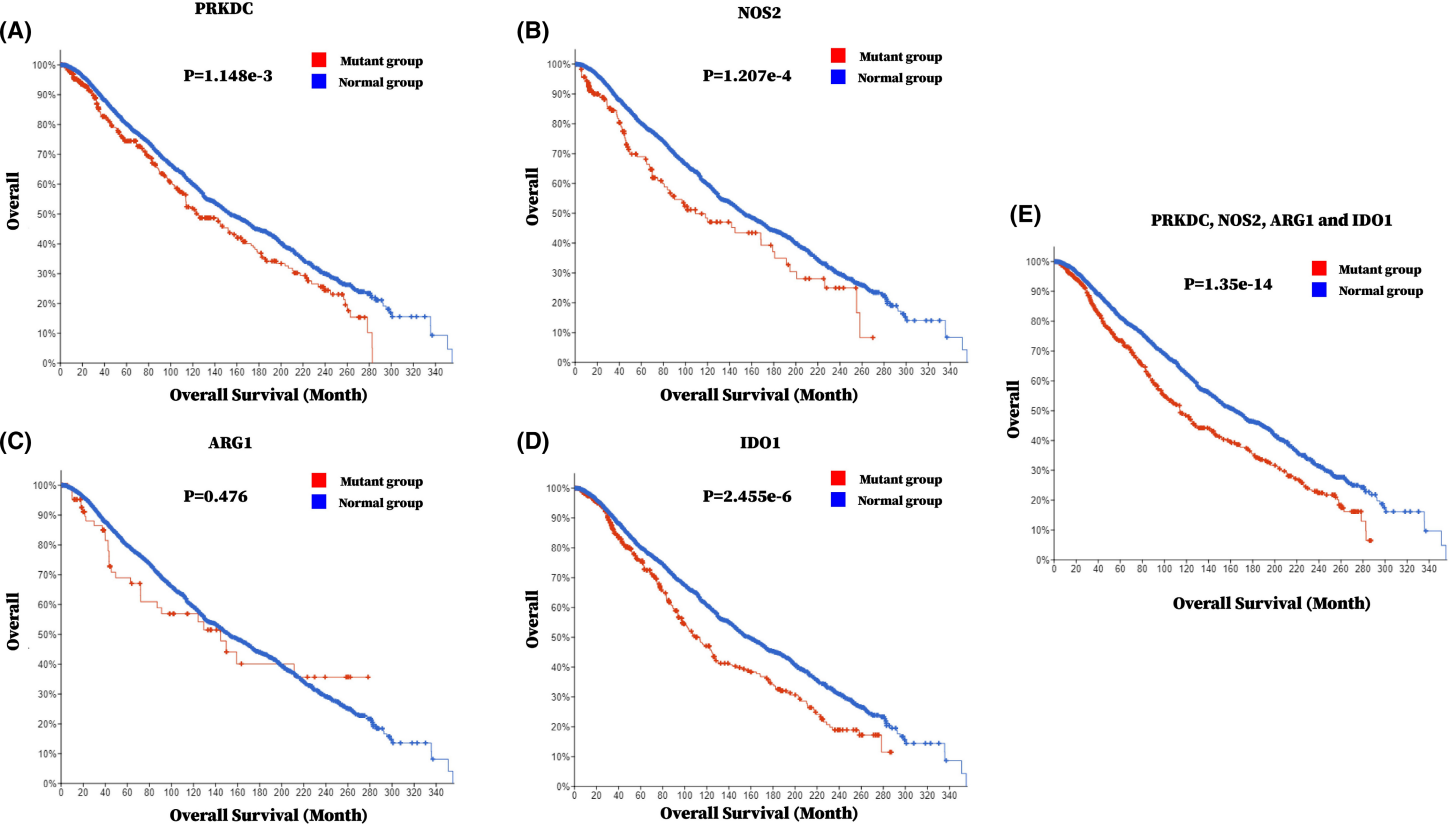
Mutations in PRKDC and functional markers of MDSCs (*iNOS*, *Arg1*, *IDO*) were associated with lower overall survival time in breast cancer patients. Analysis of the overall survival time in breast cancer patients with at least one gene mutation in *PRKDC*, *NOS2*, *Arg1*, and *IDO1* (1349 samples/1338 patients) versus those without mutations in all four genes (8206 samples/7806 patients) by cBioPortal in 19 breast cancer studies involving 9134 patients/9555 samples.

To further elucidate the important role of DNA‐PK and MDSC immunosuppressive function in breast cancer progression, we conducted a study on the correlation between mutations of DNA‐PK and functional markers of MDSCs (*iNOS*, *Arg1*, and *IDO*) and the survival of breast cancer patients. Based on cBioportal analysis, we found that the overall survival time of breast cancer patients with one gene mutation in PRKDC, NOS2, ARG1, or IDO1 was significantly shorter compared with patients without the mutations (Figure 2A–D). Besides, the overall survival time of breast cancer patients with at least one gene mutation in PRKDC, NOS2, ARG1, and IDO1 (1191 patients) was significantly shorter compared with patients without the mutations (4575 patients) (Figure 2E). This indicated that mutations in *PRKDC*, *NOS2*, *ARG1*, and *IDO1* were correlated to lower overall survival in breast cancer patients. *NOS2*, *ARG1*, and *IDO1* were the encoding genes of iNOS, Arg1, and IDO1, respectively. These proteins are the key factors in the immunosuppressive function of MDSCs. Therefore, the results suggested that DNA‐PK and MDSC immunosuppressive functions are correlated to lower overall survival in breast cancer patients.

### 3.3 NU7441 significantly reduced the proportion of MDSCs in PBMCs of tumor‐bearing mice

**FIGURE 3:**
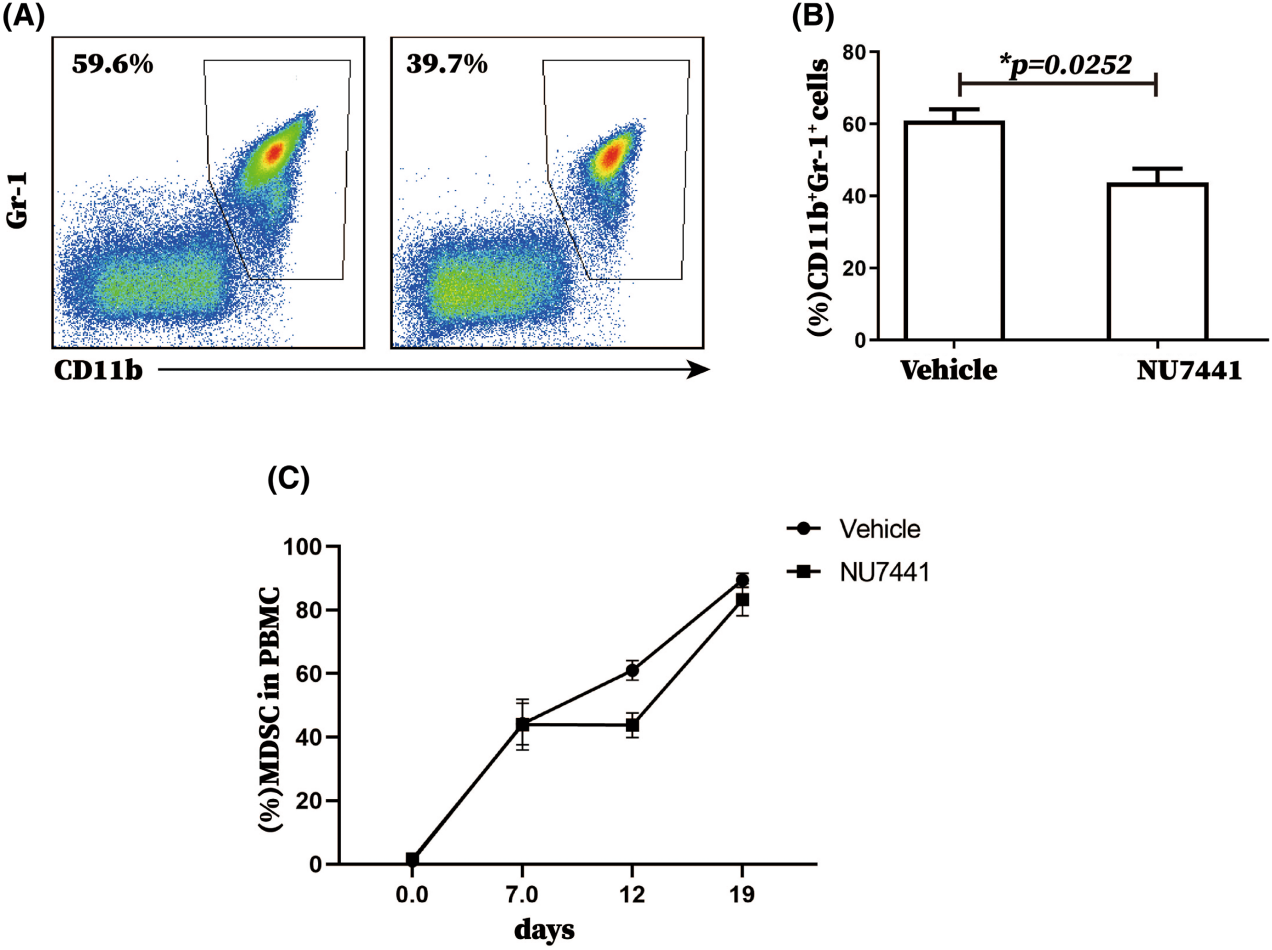
NU7441 significantly reduced the proportion of MDSCs in PBMCs of tumor‐bearing mice. Tumor‐bearing mice were divided to 2 groups with 5 mice per group and intraperitoneally injected with DMSO or NU7441 at a dose of 10 mg/kg once a day for 5 consecutive days starting on the 7th day after tumor‐bearing. Then, mandibular blood was collected. (A) Flow cytometry was used to analyze the proportion of MDSCs in the PBMCs of tumor‐bearing mice in the NU7441 treatment group and the control group, representative flow cytograms from 3 independent experiments were shown, and (B) data from 3 independent experiments were analyzed. *p < 0.05; **p < 0.01; ***p < 0.001. The experiment was repeated three times, and the data were expressed in the form of (mean ± SEM).

Next, the impact on the proportion of MDSCs was examined. The effect of NU7441 on MDSC level in PBMCs of tumor‐bearing mice by using Flow cytometry. Surprisingly, MDSC level was significantly reduced (Figure 3). In addition, we detect the number of regulatory T cells (Tregs) and CD3^+^ T cells in tumor‐bearing mice treated with NU7441, which are tightly associated with MDSCs in PBMCs of tumor‐bearing mice. Results indicated that the proportions of Tregs in CD4^+^ T cells (Figure S3A,B) and of CD3^+^ T cells in lymphocytes (Figure S4A,B) in PBMCs of tumor‐bearing mice treated with NU7441 were also significantly reduced. It was also noted that the levels of PMN‐MDSCs and Mo‐MDSCs subsets in MDSCs and the levels of CD4^+^ T and CD8^+^ T subsets in CD3^+^ T cells did not change in PBMCs of tumor‐bearing mice treated with NU7441 (Figure S5A–D).

### 3.4 NU7441 significantly enhanced the immunosuppressive function of splenic MDSCs in tumor‐bearing mice

**FIGURE 4:**
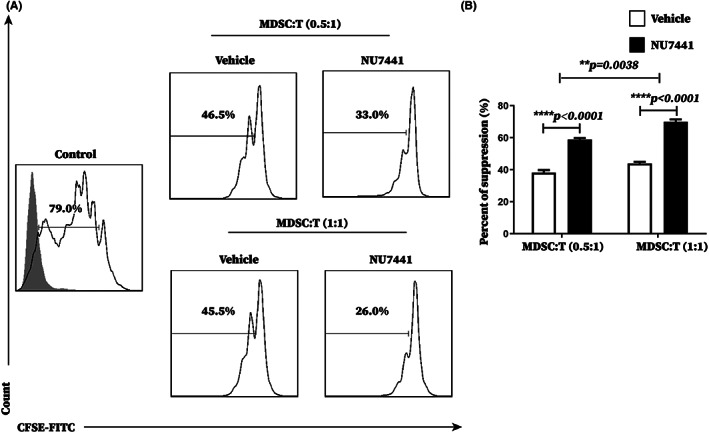
NU7441 significantly enhanced the immunosuppressive function of splenic MDSCs from tumor‐bearing mice in vivo. Tumor‐bearing mice were divided to 2 groups with 5 mice per group and intraperitoneally injected with DMSO or NU7441 at a dose of 10 mg/kg once a day for 5 consecutive days starting on the 7th day after tumor‐bearing. Tumor‐bearing mice and tumor‐bearing mice treated with NU7441 were sacrificed on the 18th day after tumor‐bearing. Spleens of tumor‐bearing mice and tumor‐bearing mice treated with NU7441 were extracted and splenic MDSCs were sorted out. (A) The inhibition test of MDSCs on T cell proliferation was used to analyze the effect of NU7441 on the immunosuppressive function of splenic MDSCs from tumor‐bearing mice in vivo, representative flow cytograms from 3 independent experiments were shown, and (b) the data from 3 independent experiments were analyzed. *p < 0.05; **p < 0.01; ***p < 0.001. The experiment was repeated three times, and the data were expressed in the form of (mean ± SEM).

Since the proportion of MDSCs in PBMCs of tumor‐bearing mice treated with NU7441 was unexpectedly reduced, we suspected NU7441 played a role in enhancing MDSC immunosuppressive function rather than in increasing the number of MDSCs. Therefore, the effect of NU7441 on immunosuppressive function of splenic MDSCs in tumor‐bearing mice was further evaluated. According to the results of proliferation inhibition assay, NU7441 administration enhanced the immunosuppressive function of splenic MDSCs of tumor‐bearing mice in vivo (Figure 4A,B) and in vitro (Figure S6A,B).

### 3.5 NU7441 significantly increased the protein levels of DNA‐PK and pDNA‐PK in MDSCs, PMN‐MDSCs, and Mo‐MDSCs of PBMCs from tumor‐bearing mice

The effect of NU7441 on DNA‐PK activity of MDSCs in tumor‐bearing mice was examined to further explore the ability of NU7441 to enhance the MDSC immunosuppressive function in tumor‐bearing mice. Firstly, the DNA‐PK and pDNA‐PK levels in MDSCs of tumor‐bearing mice PBMCs were detected by flow cytometry and analysis revealed that DNA‐PK levels in MDSCs, PMN‐MDSCs, and Mo‐MDSCs in tumor‐bearing mice PBMCs were significantly lower than those in healthy mice (Figure S7A,C,E). It also suggested that pDNA‐PK levels in MDSCs, PMN‐MDSCs, and Mo‐MDSCs in tumor‐bearing mice PBMCs were significantly higher than those in healthy mice (Figure S7B,D,F). Phosphorylation of DNA‐PK, a key promoter of the NHEJ pathway of DSB, was found to be positively correlated with DNA‐PK activity. Therefore, DNA‐PK activity in MDSCs, PMN‐MDSCs, and Mo‐MDSCs was significantly increased in PBMCs of tumor‐bearing mice compared with PBMCs of healthy mice.

**FIGURE 5:**
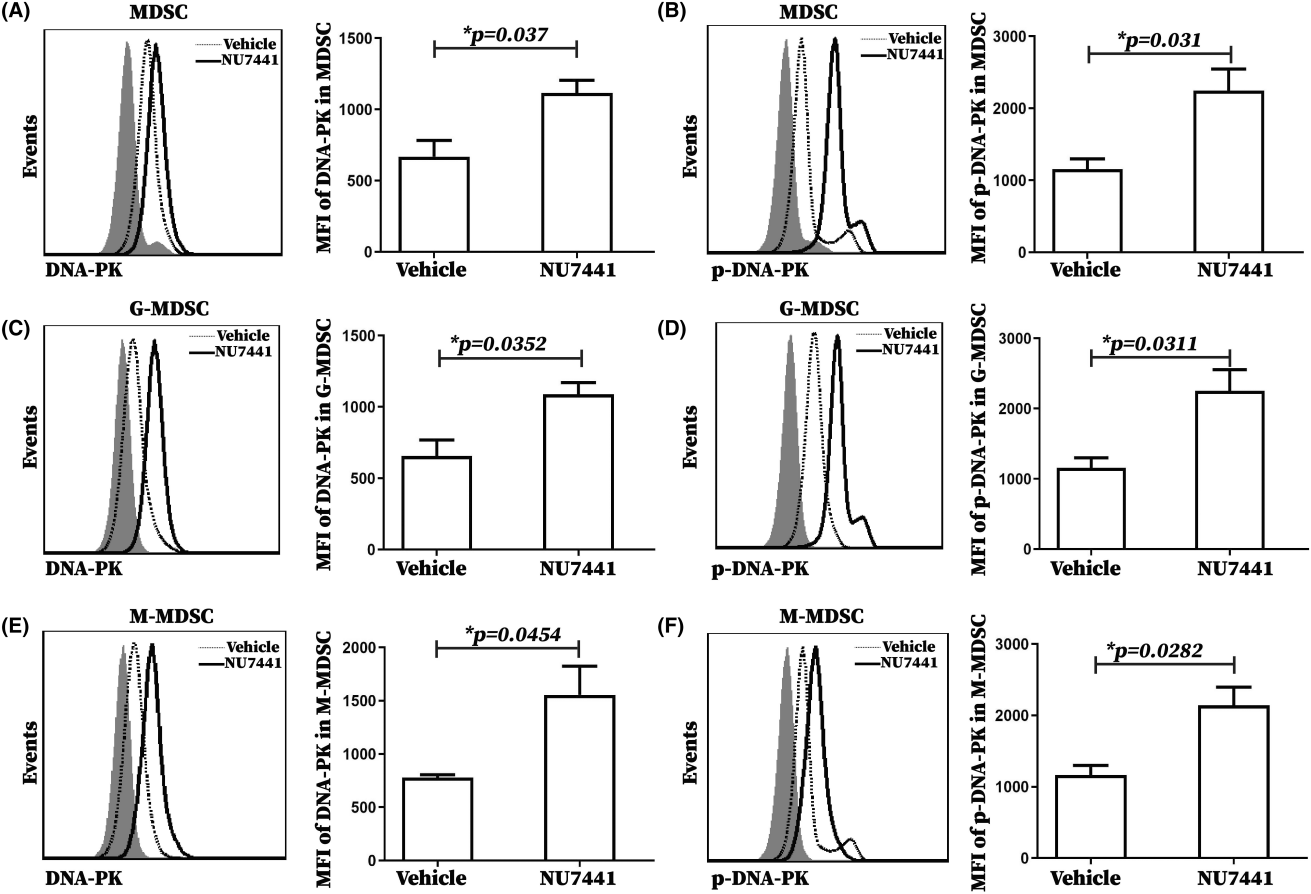
NU7441 increased the protein levels of DNA‐PK and pDNA‐PK of MDSCs, PMN‐MDSCs and Mo‐MDSCs subgroups in tumor‐bearing mice PBMCs in vivo. Tumor‐bearing mice were divided to 2 groups with 5 mice per group and intraperitoneally injected with DMSO or NU7441 at a dose of 10 mg/kg once a day for 5 consecutive days starting on the 7th day after tumor‐bearing. Then mandibular blood was collected from tumor‐bearing mice and tumor‐bearing mice treated with NU7441 and PBMCs were isolated. DNA‐PK protein levels of (A) MDSCs, (B) PMN‐MDSCs, and(C)Mo‐MDSCs in PBMCs of tumor‐bearing mice and tumor‐bearing mice treated with NU7441 were analyzed by flow cytometry and the data were statistically analyzed. pDNA‐PK protein levels of (D) MDSCs, (E) PMN‐MDSCs, and(F) Mo‐MDSCs in PBMCs of tumor‐bearing mice and tumor‐bearing mice treated with NU7441 were analyzed by flow cytometry and the data were statistically analyzed.*p < 0.05; **p < 0.01; ***p < 0.001. The experiment was repeated three times, and the data were expressed in the form of (mean ± SEM). Representative flow cytograms from 3 independent experiments were shown, and statistical histograms were quantified from 3 independent experiments.

The effects of NU7441 on the protein levels of DNA‐PK and pDNA‐PK in MDSC, PMN‐MDSCs, and Mo‐MDSCs in PBMCs of tumor‐bearing mice were further evaluated by flow cytometry analysis. In contrast to NU7441's role in inhibiting DNA‐PK phosphorylation in 4 T1 cells, we found that NU7441 administration significantly increased the protein levels of DNA‐PK and pDNA‐PK in MDSC, PMN‐MDSCs, and Mo‐MDSCs in PBMCs of tumor‐bearing mice in vivo (Figure 5A–F). Besides, NU7441 administration significantly increased the protein levels of DNA‐PK and pDNA‐PK in MDSCs, PMN‐MDSCs, and Mo‐MDSCs in tumor‐bearing mouse splenic cells in vitro (Figure S8A–F). Therefore, NU7441 significantly increased DNA‐PK activity in MDSCs, PMN‐MDSCs, and Mo‐MDSCs of tumor‐bearing mice.

### 3.6 NU7441 significantly increased the mRNA expression of DNA‐PK and functional markers of MDSCs (iNOS, Arg1, IDO) in splenic MDSCs of tumor‐bearing mice

**FIGURE 6:**
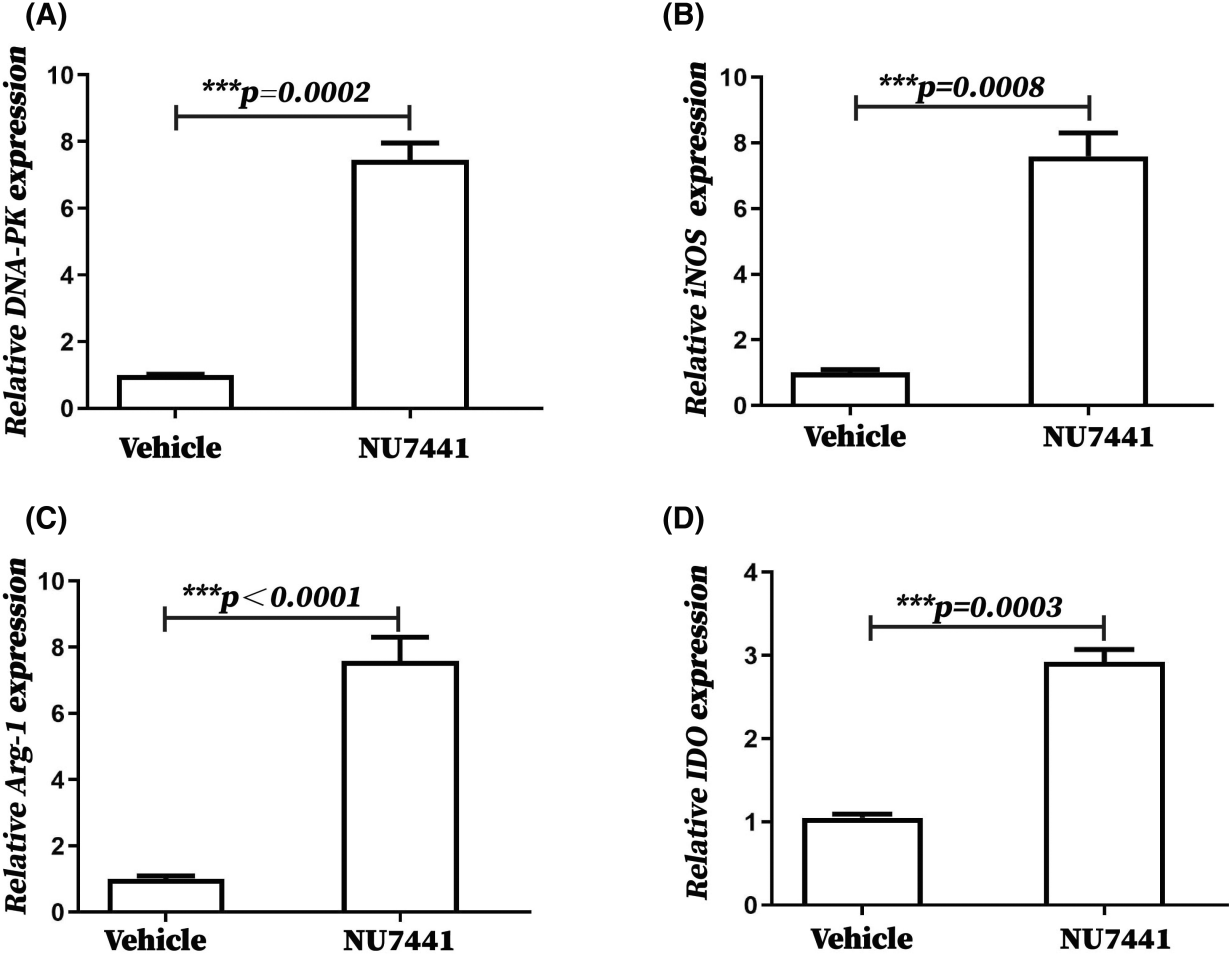
NU7441 significantly increased the mRNA expression of DNA‐PK and functional markers of MDSCs (*iNOS*, *Arg1*, *IDO*) in splenic MDSCs from tumor‐bearing mice in vitro. On the 18th day after tumor‐bearing, tumor‐bearing mice were sacrificed where their spleens were removed and splenic MDSCs were sorted out and cultured in RPMI 1640 complete medium containing 1 μmol/L NU7441 for 16 h. After RNA extraction, qPCR was used to detect the expression of (A) DNA‐PK and MDSCs functional markers (B) *iNOS*, (C) *Arg1*, and (D) *IDO* in splenic MDSCs from 5 tumor‐bearing mice treated or untreated with NU7441 in vitro. *p < 0.05; **p < 0.01; ***p < 0.001. The experiment was repeated three times, and the data were expressed in the form of (mean ± SEM). The results were quantified from 3 independent experiments.

The effects of NU7441 on the mRNA expression of DNA‐PK and functional markers of MDSCs (*iNOS*, *Arg1*, and *IDO*) in splenic MDSCs of tumor‐bearing mice were detected using qPCR. First, our results showed that DNA‐PK expression was significantly increased in splenic MDSCs of tumor‐bearing mice compared with that in healthy control mice (Figure S9), which was consistent with previous researches that patients with breast cancer showed higher PRKDC (DNA‐PK coding gene) expression than healthy controls. Interestingly, the expression of DNA‐PK and functional markers of MDSCs (*iNOS*, *Arg1*, and *IDO*) were significantly increased in splenic MDSCs of tumor‐bearing mice treated with NU7441 in vitro (Figure 6A–D), suggesting that NU7441 significantly increased the expression of DNA‐PK and the immunosuppressive function of MDSCs. In addition, to obtain a more accurate conclusion, we also detected the expression of DNA‐PK and MDSCs functional markers (*iNOS*, *Arg1*, and *IDO*) in splenic MDSCs of tumor‐bearing mice treated with NU7441 in vivo. The expression of DNA‐PK and functional markers of MDSCs (*iNOS*, *Arg1*, and *IDO*) in splenic MDSCs from tumor‐bearing mice treated with NU7441 in vivo was significantly increased (Figure S10A–D), which was consistent with the results of NU7441 administration in vitro.

### 3.7 NU7441 combined with gemcitabine significantly reduced tumor volume by reducing the proportion of MDSCs in tumor‐bearing mice

**FIGURE 7:**
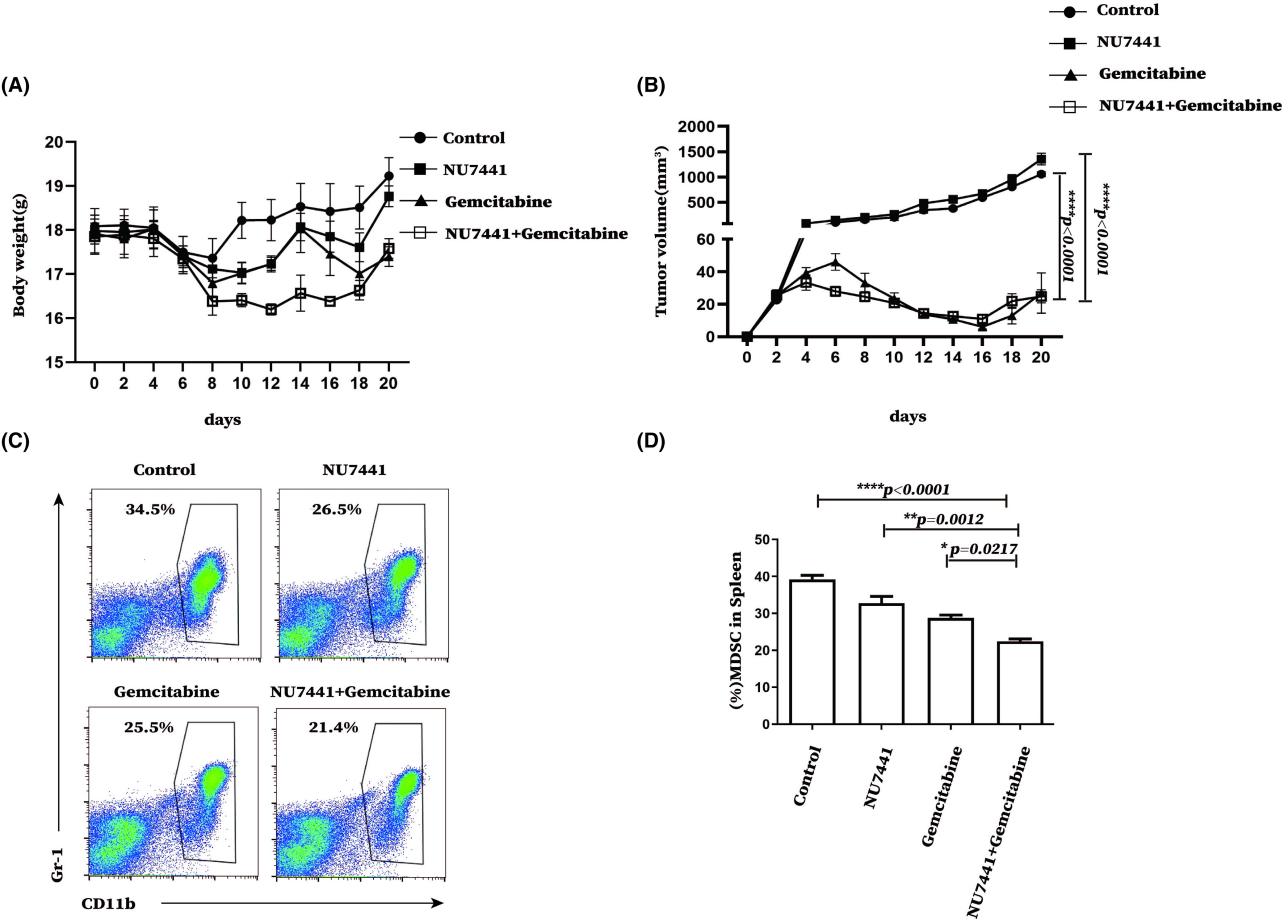
NU7441 combined with Gemcitabine significantly reduced tumor volume and the proportion of splenic MDSCs from tumor‐bearing mice. After tumor‐bearing, the mice were divided to 4 groups with 5 mice per group and intraperitoneally injected with DMSO or NU7441 at a dose of 10 mg/kg for 5 consecutive days from day 8, and Gemcitabine at a dose of 100 mg/kg was injected intraperitoneally on days 3, 6, 9, and 12. (A) The body weight of the mice was recorded and the body weight change curve of the mice was plotted from 3 independent experiments. (B) Tumor volume of mice was recorded and the curve of tumor volume was plotted from 3 independent experiments. After NU7441 and Gemcitabine were given, the mice were sacrificed at day 20, and their spleens were removed to prepare a single‐cell suspension. (C) The proportion of splenic MDSCs from tumor‐bearing mice was analyzed by flow cytometry, representative flow cytograms from 3 independent experiments were shown. and (D) the data were statistically analyzed from 3 independent experiments. *p < 0.05; **p < 0.01; ***p < 0.001. The experiment was repeated three times, and the data were expressed in the form of (mean ± SEM).

The abovementioned results suggested that NU7441 administration could enhance the DNA‐PK activity in MDSCs and thus promote the immunosuppressive function, and ultimately tumor growth. Therefore, whether the removal of MDSCs could reverse the tumor‐promoting effect of NU7441 was explored. Here, NU7441 was combined with gemcitabine, a widely used chemotherapy drug to remove MDSCs, in treating tumor‐bearing mice and its effect was observed. Firstly, we found that NU7441, gemcitabine, and NU7441 combined with gemcitabine had no significant effect on the body weight of tumor‐bearing mice (Figure 7A). Additionally, we found that NU7441 combined with gemcitabine significantly reduced tumor volume during administration and at the beginning of drug withdrawal (Figure 7B).

Given that gemcitabine is a commonly used chemotherapy drug which can effectively remove MDSCs, and NU7441 administration in the above results can also reduce the number of MDSCs. Based on the results above, it was hypothesized that NU7441 combined with gemcitabine can reduce tumor volume by effectively removing MDSCs. Flow cytometry analysis revealed that NU7441 combined with gemcitabine significantly reduced the proportion of MDSCs in tumor‐bearing mice PBMCs (Figure S11A,B), the level of splenic MDSCs from tumor‐bearing mice (Figure 7C,D) and the level of tumor infiltrating MDSCs (Figure S12A,B). In addition, results revealed that the combination could significantly reduce the proportion of Tregs in CD4^+^ T cells (Figure S13A,B) and also increase the proportion of CD3^+^ T cells in splenic lymphocytes of tumor‐bearing mice (Figure S14A,B). Furthermore, the combination did not affect the proportion of CD4^+^ T cells and CD8^+^ T cells in CD3^+^ T cells in the spleen of tumor‐bearing mice (Figure S15A–C).

## 4 DISCUSSION

TNBC can still threat the patient survival due to its high heterogeneity, strong aggression, lack of receptors, shortage of treatment options, strong resistance to chemotherapy, and poor clinical prognosis.^33^ MDSCs were significantly increased in TNBC patients and affect the TNBC progress.^12^ So, the regulation of MDSCs gives a new direction for future treatment of TNBC. DNA‐PK is overexpressed in breast cancer, including TNBC.^21^, ^22^ It was closely related to poor prognosis of breast cancer,^21^ which indicates that it is a new therapeutic target for breast cancer, including TNBC. At present, DNA‐PK inhibition is commonly used in tumor treatment in preclinical trials, while DNA‐PK inhibitors are usually only used as sensitizers of radiotherapy and chemotherapy but are ineffective when used alone in preclinical trials of tumor treatment.^27^, ^28^ The specific reasons remain unclear. NU7441 is found involved in the regulation of CD4^+^ T cell function and differentiation,^29^ and can also be used to enhance the immunogenicity of moDC,^30^ but the effect of NU7441 on MDSCs has not been studied. Our study was groundbreaking to demonstrate that NU7441 could significantly improve the DNA‐PK activity in MDSCs thus enhance MDSC immunosuppressive function, further significantly promoting TNBC progression. We also firstly provide possible reasons why NU7441 is effective when used in combination with other therapies and not effective when used alone. These results help to better understand the function of NU7441 and will become more valuable as NU7441 therapies are developed.

The results in tumor‐bearing mice model showed that NU7441 significantly promoted the progression of TNBC, both in terms of tumor volume and weight. Previous animal models of human colon cancer SW620 xenograft showed that NU7441 increased the cytotoxicity of etoposide to slow down tumor progression; however, NU7441 alone did not show a significant effect.^34^ The use of NU7441 alone in our study promoted the progression of TNBC, which may be caused by the difference in MDSCs levels between TNBC and colon cancer. Furthermore, in 4 T1 cells, we found that NU7441 significantly reduced pDNA‐PK protein levels without affecting DNA‐PK protein levels and its mRNA expression. This is consistent with the results reported by other studies that NU7441 inhibited DNA‐PK phosphorylation in uveal melanoma cells without affecting DNA‐PK protein levels,^35^, ^36^ suggesting that NU7441 plays a part in inhibiting DNA‐PK phosphorylation in 4 T1 cells. The different effects of NU7441 on 4 T1 cells and tumors in tumor‐bearing mice prompted the exploration of the mechanism through which NU7441 promoted tumor growth in tumor‐bearing mice.

Here, we proved that DNA‐PK was correlated to MDSC function and with breast cancer survival. Our results showed that DNA‐PK tended to be co‐expressed with various functional markers of MDSCs (*iNOS*, *Arg1*, and *IDO*), and that the overall survival time of breast cancer patients with mutations of DNA‐PK and various functional markers of MDSCs (*iNOS*, *Arg1*, and *IDO*) was significantly reduced. This means that regulating DNA‐PK and the functions of MDSCs can serve as a breast cancer treatment option.

We also demonstrated that NU7441 significantly reduced the proportion of MDSCs, related Tregs, and CD3^+^ T cells in tumor‐bearing mice PBMCs. However, it did not affect the proportion of PMN‐MDSCs and Mo‐MDSCs subsets in MDSCs as well as the proportion of CD4^+^ T and CD8^+^ T cell subsets in CD3^+^ T cells. Consistent to previous researches, the number of Tregs was affected by the number of MDSCs^37^ and that the number of CD3^+^ T is inversely proportional to MDSCs function.^38^ It is also possible that NU7441 inhibited the repair function of DNA‐PK, leading to the death of MDSCs, Tregs, and CD3^+^ T cells. Instead of thinking that tumor growth was promoted due to the increase in number of MDSCs, it is possible that NU7441 may have promoted tumor growth by enhancing the MDSCs function.

It was also demonstrated that NU7441 enhanced the immunosuppressive function of splenic MDSCs of tumor‐bearing mice in vivo and in vitro, which was consistent with our qPCR results, suggesting that NU7441 could indeed enhance MDSC immunosuppressive function.

Some studies have suggested that reduced DNA‐PK activity could lead to cancer because DNA‐PK is used to repair defects, while higher DNA‐PK expression and activity has been observed in other tumor cells and is correlated with reduced efficiency of anti‐tumor drugs.^39^ In this study, it was found that DNA‐PK levels in MDSCs, PMN‐MDSCs, and Mo‐MDSCs in tumor‐bearing mice PBMCs were lower than those in healthy mice, while pDNA‐PK (a post‐translational modification positively correlated with the kinase activity of DNA‐PK) levels were higher, which was consistent with the latter conclusion. However, results also showed that NU7441 could significantly increase the levels of DNA‐PK and pDNA‐PK of MDSCs, PMN‐MDSCs, and Mo‐MDSCs subgroups in PBMCs and spleen of tumor‐bearing mice. This is consistent with our qPCR results, but contrary to the previous studies that found that NU7441 significantly inhibited the phosphorylation of DNA‐PK in uveal melanoma cells without affecting DNA‐PK protein levels,^35^, ^36^ which further proved our hypothesis. In conclusion, NU7441 played a role in promoting DNA‐PK activity in tumor‐bearing mice MDSCs.

Furthermore, we found that NU7441 increased the mRNA expression of DNA‐PK and functional markers of MDSCs (*iNOS*, *Arg1*, *IDO*) in the splenic MDSCs of tumor‐bearing mice both in vivo and in vitro. We also suggested that the expression of DNA‐PK in splenic MDSCs of tumor‐bearing mice was higher than that of healthy mice, which was consistent with the high expression of DNA‐PK in TNBC in previous studies.^39^ Nevertheless, there was no difference in DNA‐PK expression in 4 T1 cells treated with NU7441, which is consistent to previous studies that NU7441 alone was ineffective.^40^, ^41^ In addition, our qPCR results suggested that NU7441 could increase the mRNA expression of DNA‐PK and functional markers of MDSCs (*iNOS*, *Arg1*, *IDO*) in the splenic MDSCs of tumor‐bearing mice in vivo and in vitro suggesting that the effect of NU7441 alone on DNA‐PK in tumor‐bearing mice MDSCs may be different from previous studies. This may also suggest that NU7441 may enhance the function of MDSCs, which may be the reason behind its tumor‐promoting effect instead of tumor inhibition effect.

Combination of NU7441 and gemcitabine also reduced tumor volume in tumor‐bearing mice by effectively removing MDSCs. This was consistent with previous studies that this combination significantly reduced tumor volume in tumor‐bearing mice with lung cancer.^42^ Further studies showed that this combination reduced the proportion of MDSCs in PBMCs, spleen and tumor microenvironment of tumor‐bearing mice, which may be caused by the effect of NU7441 on reducing the level of MDSCs and gemcitabine's potential in removing MDSCs in tumor‐bearing mice. Furthermore, it was also demonstrated that the combination reduced the proportion of MDSCs‐related Tregs in the spleen of tumor‐bearing mice, which was consistent with the previous findings that the number of Tregs was affected by the number of MDSCs.^37^ Increased proportion of CD3^+^ T cells associated with MDSCs in spleen of tumor‐bearing mice was also observed, but it did not affect the proportions of CD4^+^ T and CD8^+^ T cell subsets in CD3^+^ T cells. This may be because the overall immunosuppressive function of MDSCs was weakened leading to the increase in proportion of CD3^+^ T cells, which is consistent to previous findings that the number of CD3^+^ T cells was inversely proportional to MDSC function.^38^

Since the specific mechanism behind the reduction in number and the enhancement of MDSC's function through the NU7441‐mediated regulation of DNA‐PK activity remains unclear, we can reasonably speculate that this might be due to the compensatory effect of NU7441 reducing the number of MDSC. NU7441 reduced the number of MDSC but enhanced the DNA‐PK activity of the remaining MDSC, thus enhancing the overall immunosuppressive function of MDSC and promoting tumor growth. This can be a good avenue for future research to focus on. Additionally, future research should focus on the effect of the combination of NU7441 and gemcitabine on the development of therapies for TNBC.

In summary, we demonstrated that NU7441‐mediated regulation of DNA‐PK activity promoted tumor progression in tumor‐bearing mice, which was dependent upon the significant enhancement of the immunosuppressive function of MDSCs. At the same time, our data suggested a possible explanation why the use of NU7441 alone in previous studies had no therapeutic effect on tumors. In addition, the combination of NU7441 and gemcitabine was found to be effective in removing MDSCs and in reducing tumor size.

## AUTHOR CONTRIBUTIONS

**Jiawen HAN:** Writing – original draft (lead). **Minjie Wan:** Investigation (equal). **Zhanchuan Ma:** Visualization (equal). **Huanfa Yi:** Supervision (lead); writing – review and editing (lead).

## FUNDING INFORMATION

This work was supported by grants from the National Natural Science Foundation of China (81671592) and the Science and Technology Department of Jilin Province (20190201140JC).

## CONFLICT OF INTEREST

The authors declare no conflicts of interest.

## ETHICS STATEMENT

The animal study was reviewed and approved by the Subcommittee on Research Animal Care of the First Hospital of Jilin University.

## Supporting information


Appendix S1

## Data Availability

The raw data supporting the conclusions of this article will be made available by the authors, without undue reservation.
