# Supplementary material for: Regulation of DNA‐PK activity promotes the progression of TNBC via enhancing the immunosuppressive function of myeloid‐derived suppressor cells
Source: Cancer Med. 2022 Nov 13;12(5):5939–52. doi: 10.1002/cam4.5387 (PMC10028116; doi:10.1002/cam4.5387)
Supplement: Supplementary file 1 — Appendix S1 [file CAM4-12-5939-s001.docx]

**Supplementary Data**

Supplementary Table 1．Oligonucleotide and primer sequence

| Primer name | Primer sequence |
| --- | --- |
| *β-actin* | F: 5’ TTCACACCCCAGCCATG 3’  R: 5’ CCTCGTAGATGGGCACAGT 3’ |
| *Arg-1* | F:5’TGTCCCTATGACAGCTCCTT 3’  R:5’GCATCCACCCAATGACACAT 3’ |
| *iNOS* | F:5’TGGCCACCTTGTTCAGCTACG 3  R: GCCAGGCCAACACAGCATAC 3’ |
| *IDO* | F:5’ GCTTTGCTCTACCACATCCAC 3’  R:5’ CAGGCGCTGTAACCTGTGT 3’ |
| *DNA-PK* | F:5’ AAACCTGTTCCGAGCTTTTCTG 3’  R:5’ TCTCAATCTGAGGACGAATTGC 3’ |


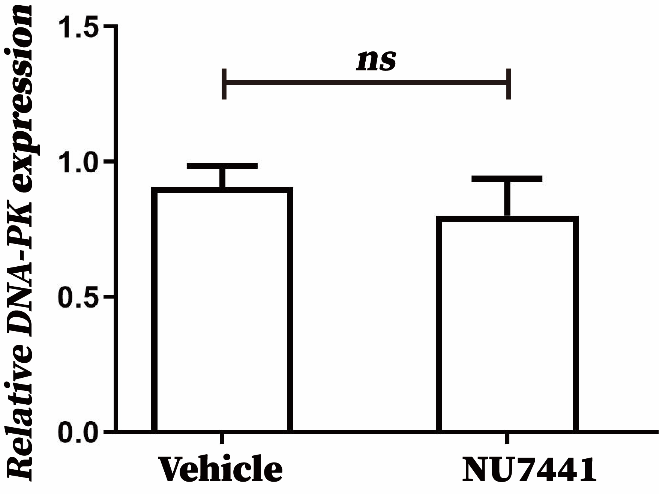


**Supplementary Figure 1. NU7441 had no significant effect on DNA-PK mRNA expression in 4T1 cells.** 4T1 was cultured in DMEM containing 1μmol/ LNU7441 for 16h. After RNA extraction, the expression of DNA-PK in NU7441-treated or untreated 4T1 cells in vitro was detected by qPCR. *P < 0.05; * * P < 0.01; * * * P < 0.001. The experiment was repeated three times, and the data were expressed in the form of (mean ±SEM). The result was quantified from 3 independent experiments.


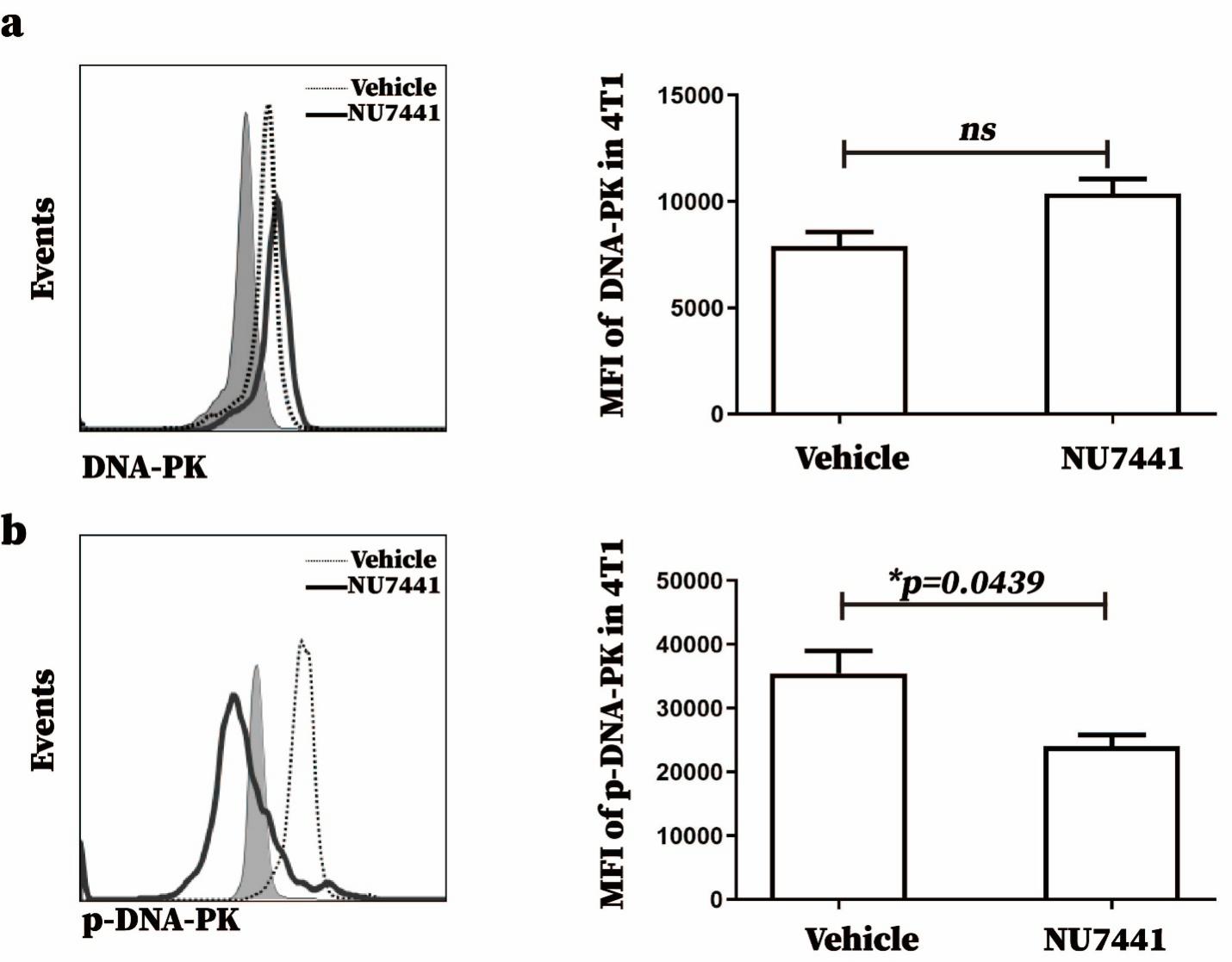


**Supplementary Figure 2. NU7441 decreased pDNA-PK protein levels in 4T1 cells without affecting DNA-PK protein levels.** 4T1 cells were cultured in complete DMEM medium with or without 1μmol/ L NU7441 for 16h, and the protein levels of (a) DNA-PK and (b) pDNA-PK in 4T1 cells treated with or without NU7441 were analyzed by flow cytometry. *P < 0.05; * * P < 0.01; * * * P < 0.001. The experiment was repeated three times, and the data were expressed in the form of (mean ±SEM). Representative flow cytograms from 3 independent experiments were shown, and statistical histograms were quantified from 3 independent experiments.


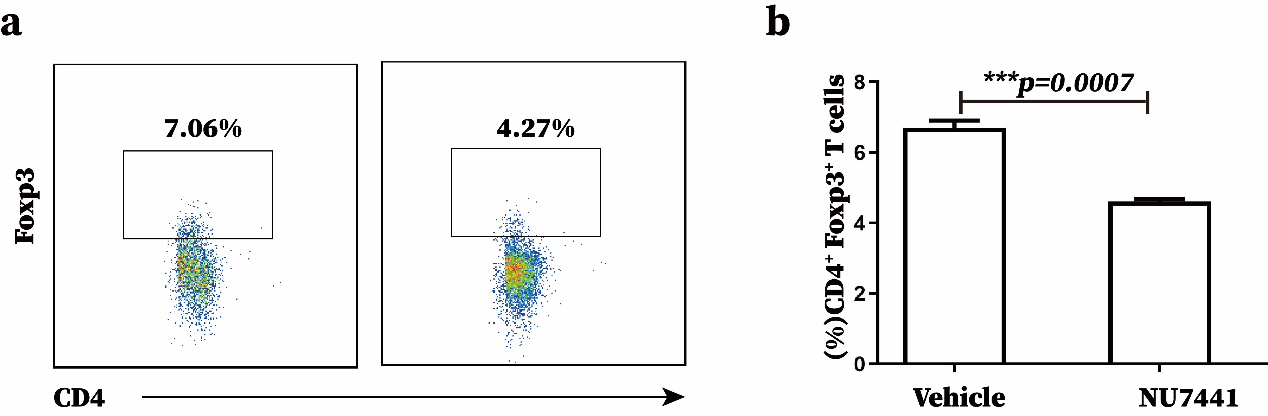


**Supplementary Figure 3. NU7441 significantly reduced the proportion of T_reg_ in CD4^+^T cells in PBMCs of tumor-bearing mice.** Tumor-bearing mice were divided to 2 groups with 5 mice per group and intraperitoneally injected with DMSO or NU7441 at a dose of 10mg/kg once a day for 5 consecutive days starting on the 7th day after tumor-bearing. Then, mandibular blood was collected. (a) Flow cytometry was used to analyze the proportion of Treg in CD4^+^T cells in PBMCs of tumor-bearing mice in the NU7441 treatment group and the control group, and (b) data were analyzed. * P < 0.05; * * P < 0.01; * * * P < 0.001. The experiment was repeated three times, and the data were expressed in the form of (mean ±SEM). Representative flow cytograms from 3 independent experiments were shown, and statistical histogram was quantified from 3 independent experiments.


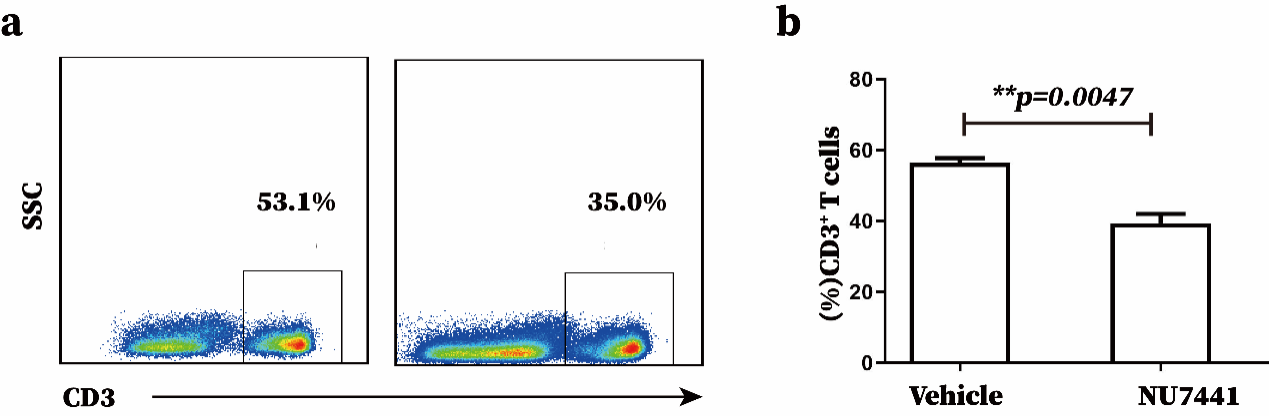


**Supplementary Figure 4. NU7441 significantly reduced the proportion of CD3^+^T cells in lymphocytes of PBMCs in tumor-bearing mice.** Tumor-bearing mice were divided to 2 groups with 5 mice per group and intraperitoneally injected with DMSO or NU7441 at a dose of 10mg/kg once a day for 5 consecutive days starting on the 7th day after tumor-bearing. Then mandibular blood was collected. (a) Flow cytometry was used to analyze the proportion of CD3^+^T cells in the PBMCs of tumor-bearing mice in the NU7441 treatment group and the control group, and (b) to perform data statistics. * P < 0.05; * * P < 0.01; * * * P < 0.001. The experiment was repeated three times, and the data were expressed in the form of (mean ±SEM). Representative flow cytograms from 3 independent experiments were shown, and statistical histogram was quantified from 3 independent experiments.


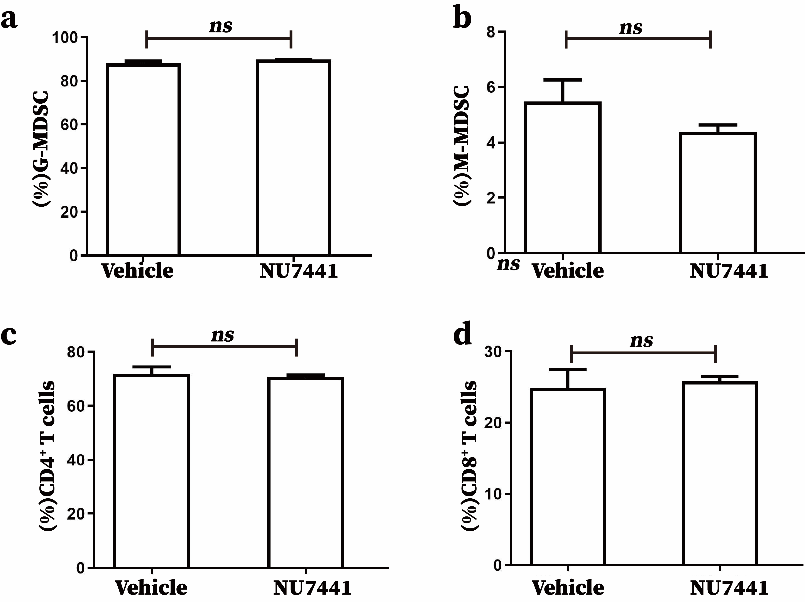


**Supplementary Figure 5. NU7441 did not affect the proportion of PMN-MDSCs and Mo-MDSCs subsets in MDSCs and the proportion of CD4^+^T and CD8^+^T cell subsets in CD3^+^T cells in PBMCs of tumor-bearing mice.** Tumor-bearing mice were divided to 2 groups with 5 mice per group and intraperitoneally injected with DMSO or NU7441 at a dose of 10mg/kg, once a day, for 5 consecutive days, on the seventh day after tumor-bearing. Then, mandibular blood was collected and flow cytometry was used to analyze the proportion of (a) PMN-MDSCs and (b) Mo-MDSCs subsets in MDSCs as well as the proportion of (c) CD4^+^T and (d) CD8^+^T subsets in CD3^+^T cells in PBMCs of tumor-bearing mice in NU7441 treatment group and control group, and the data were statistically analyzed. *P < 0.05; * * P < 0.01; * * * P < 0.001. The experiment was repeated three times, and the data were expressed in the form of (mean ±SEM). The results were quantified from 3 independent experiments.


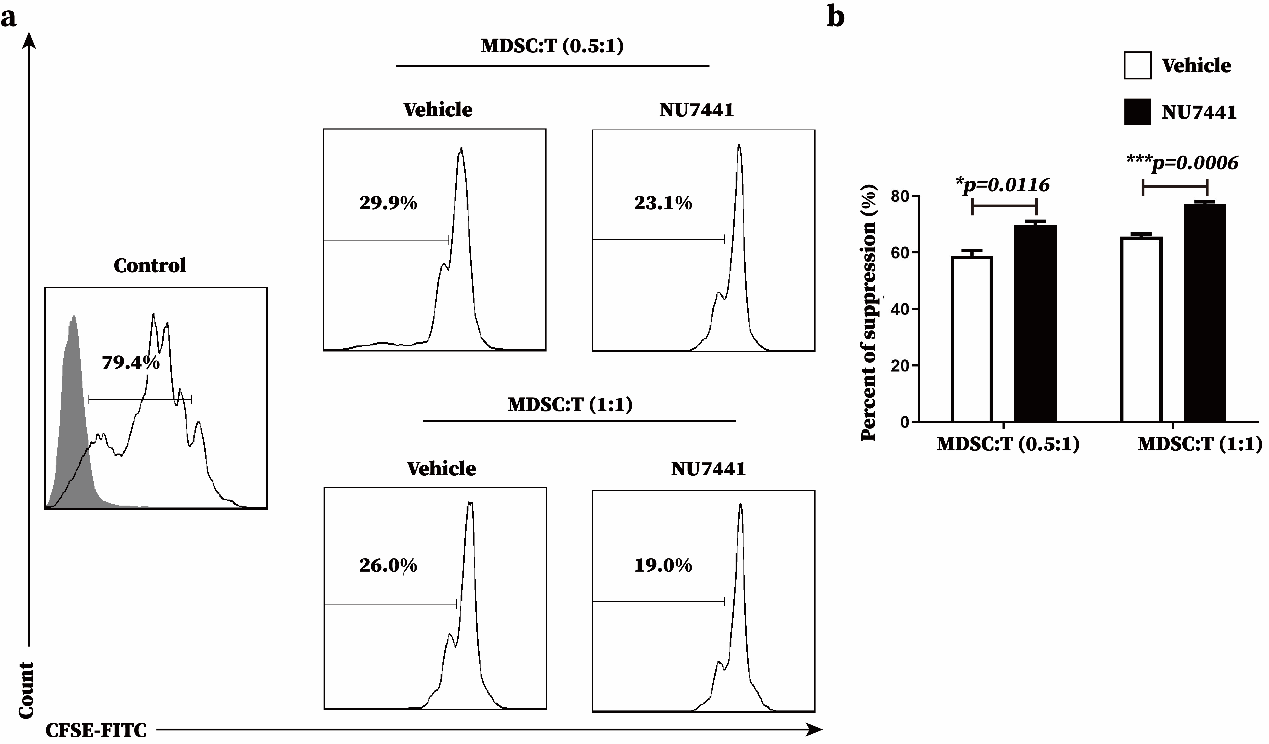


**Supplementary Figure 6. NU7441 significantly enhanced the immunosuppressive function of splenic MDSCs from tumor-bearing mice in vitro.** On the 18th day after tumor-bearing, tumor-bearing mice were sacrificed, their spleens were removed and splenic MDSCs were sort out and cultured in RPMI 1640 complete medium containing 1μmol/ L NU7441 for 16h.(a) Analyzing the effect of NU7441 on the immunosuppressive function of splenic MDSCs from 5 tumor-bearing mice in vitro by the inhibition test of MDSCs on T cell proliferation, and (b) to conduct data statistics. *P < 0.05; * * P < 0.01; * * * P < 0.001. The experiment was repeated three times, and the data were expressed in the form of (mean ±SEM). Representative flow cytograms from 3 independent experiments were shown, and statistical histogram was quantified from 3 independent experiments.


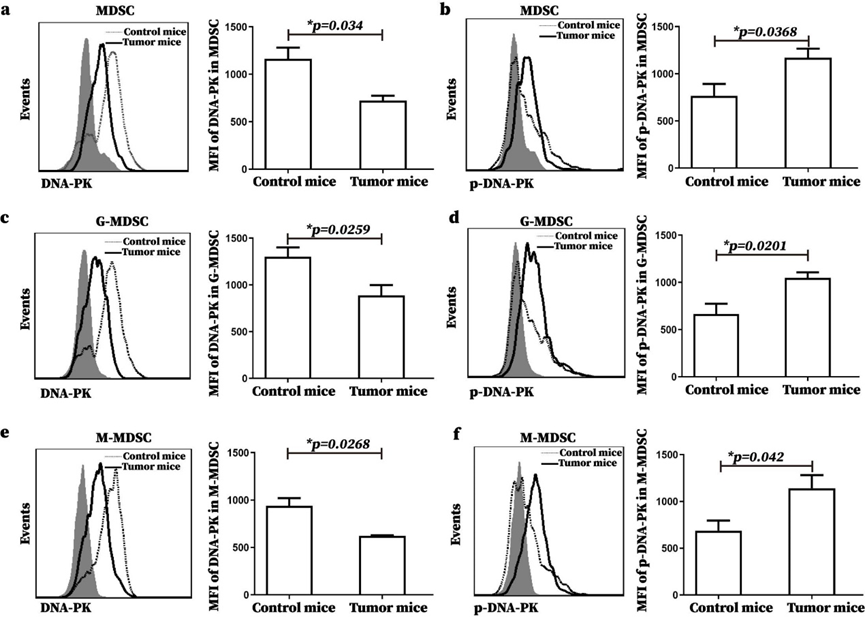


**Supplementary Figure 7. Tumor-bearing mice showed significantly lower DNA-PK protein levels in PBMCs, PMN-MDSCs, and Mo-MDSCs, while significantly higher pDNA-PK protein levels.** At the 7th day after tumor-bearing, mandibular blood was collected from 5 tumor-bearing mice and 5 healthy mice then PBMCs were isolated. DNA-PK protein levels in (a) MDSCs, (b) PMN-MDSCs, and (c) Mo-MDSCs in PBMCs of 4 tumor-bearing mice and healthy mice were analyzed by flow cytometry and the data were statistically analyzed. pDNA-PK protein levels in (d) MDSCs, (e) PMN-MDSCs and (f) Mo-MDSCs in tumor-bearing mice PBMCs and healthy mice PBMCs were analyzed by flow cytometry and the data were statistically analyzed. *P < 0.05; * * P < 0.01; * * * P < 0.001. The experiment was repeated three times, and the data were expressed in the form of (mean ±SEM). Representative flow cytograms from 3 independent experiments were shown, and statistical histograms were quantified from 3 independent experiments.


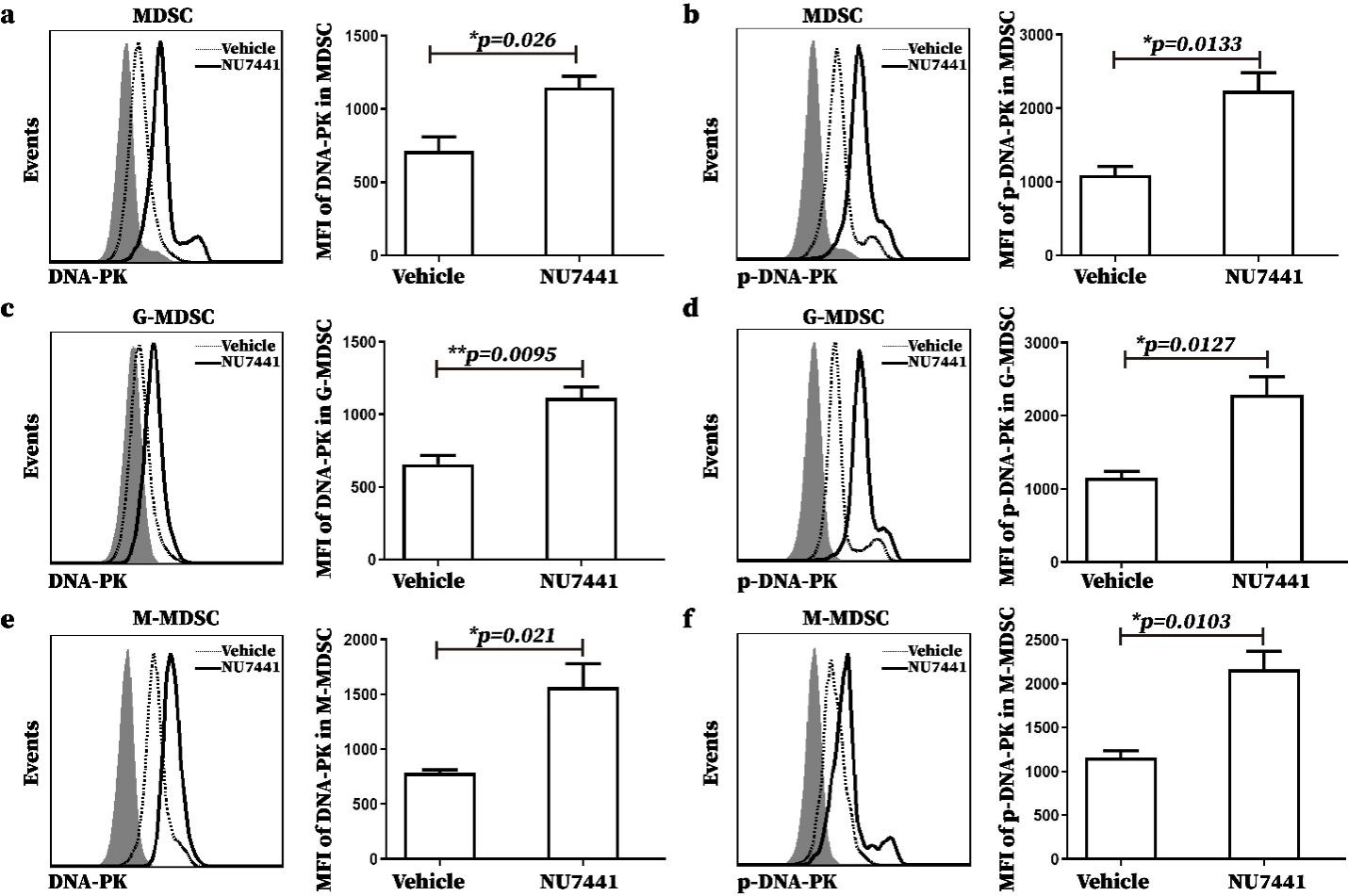


**Supplementary Figure 8. NU7441 increased the protein levels of DNA-PK and pDNA-PK in MDSCs, PMN-MDSCs and Mo-MDSCs subgroups in spleen of tumor-bearing mice in vitro.** On the 18th day after tumor-bearing, tumor-bearing mice were sacrificed, their spleens were removed and splenic MDSCs were sort out and cultured in RPMI 1640 complete medium supplemented with 1μmol/ L NU7441 for 16h. DNA-PK protein levels in splenic (a) MDSCs, (b) PMN-MDSCs and (c) Mo-MDSCs from 5 tumor-bearing mice treated or untreated with NU7441 in vitro were analyzed by flow cytometry and the data were statistically analyzed. pDNA-PK protein levels in splenic (d) MDSCs, (e) PMN-MDSCs and (f) Mo-MDSCs from 5 tumor-bearing mice treated or untreated with NU7441 in vitro were analyzed by flow cytometry and the data were statistically analyzed. *P < 0.05; * * P < 0.01; * * * P < 0.001. The experiment was repeated three times, and the data were expressed in the form of (mean ±SEM). Representative flow cytograms from 3 independent experiments were shown, and statistical histograms were quantified from 3 independent experiments.


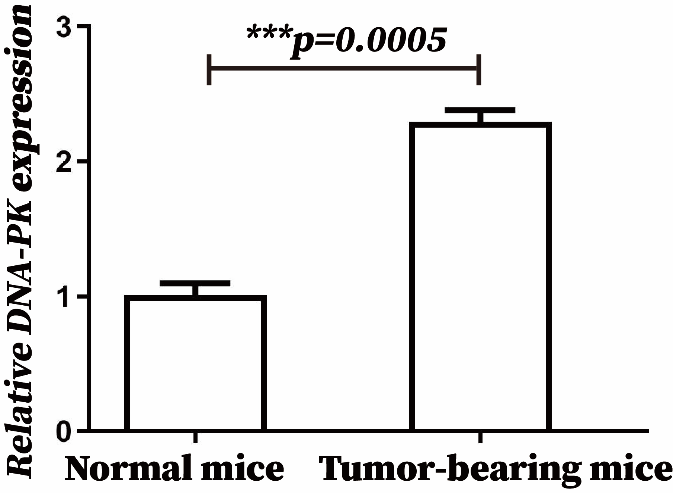


**Supplementary Figure 9. DNA-PK mRNA expression in splenic MDSCs from tumor-bearing mice was higher.** On the 18th day after tumor-bearing, 5 tumor-bearing mice and 5 healthy mice were sacrificed. Spleens of tumor-bearing mice and healthy mice were removed and splenic MDSCs were sorted out. After RNA extraction, the expression of DNA-PK in splenic MDSCs from tumor-bearing mice and healthy mice was detected by qPCR. *P < 0.05; * * P < 0.01; * * * P < 0.001. The experiment was repeated three times, and the data were expressed in the form of (mean ±SEM). The result was quantified from 3 independent experiments.


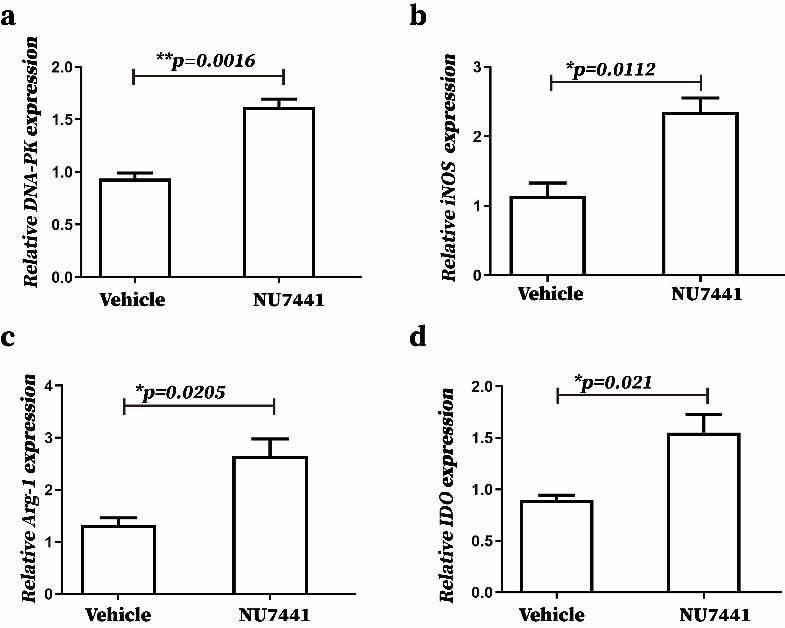


**Supplementary Figure 10. NU7441 significantly increased the mRNA expression of DNA-PK and functional markers of MDSCs (iNOS, Arg1, IDO) in splenic MDSCs from tumor-bearing mice in vivo.** Tumor-bearing mice were divided to 2 groups with 5 mice per group and intraperitoneally injected with DMSO or NU7441 at a dose of 10mg/kg once a day for 5 consecutive days on the 7th day after tumor-bearing. Tumor-bearing mice and tumor-bearing mice treated with NU7441 were sacrificed on the 18th day after tumor-bearing. Spleens of tumor-bearing mice and tumor-bearing mice treated with NU7441 were taken out and splenic MDSCs were sorted out. Then RNA was extracted and qPCR was used to detect the expression of (a)DNA-PK and functional markers of MDSCs ((b)iNOS, (c)Arg1, (d)IDO) in splenic MDSCs from tumor-bearing mice treated with NU7441 or untreated in vivo. *P < 0.05; * * P < 0.01; * * * P < 0.001. The experiment was repeated three times, and the data were expressed in the form of (mean ±SEM). The results were quantified from 3 independent experiments.


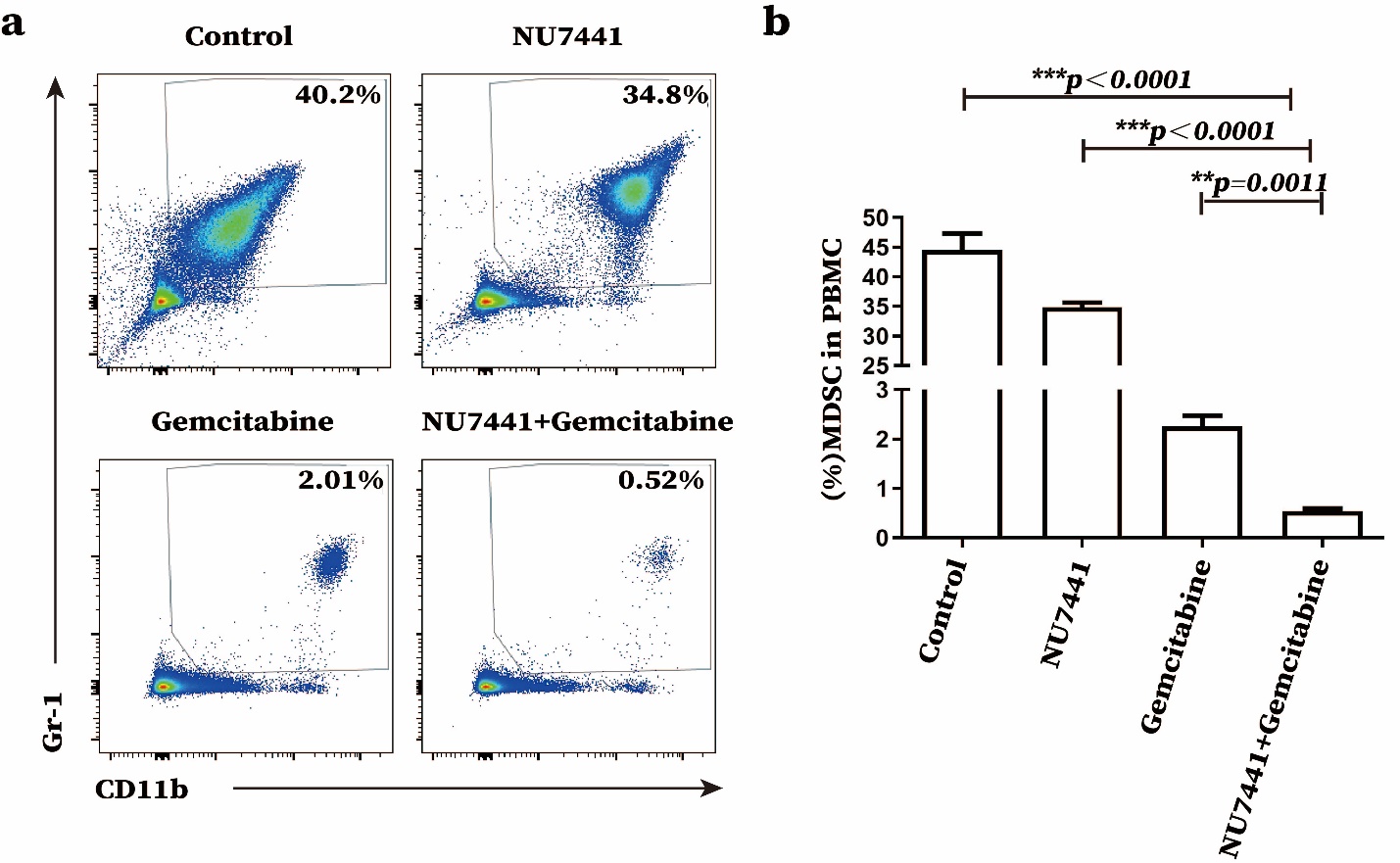


**Supplementary Figure 11. NU7441 combined with Gemcitabine significantly reduced the proportion of MDSCs in PBMCs of tumor-bearing mice.** After tumor-bearing, the mice were divided to 4 groups with 5 mice per group and intraperitoneally injected with DMSO or NU7441 at a dose of 10mg/kg for 5 consecutive days from day 8, intraperitoneally injected with Gemcitabine at a dose of 100mg/kg on day 3, day 6, day 9 and day 12, then mandibular blood was collected and PBMCs were isolated at the end of NU7441 and Gemcitabine administration.(a) The proportion of MDSCs in tumor-bearing mice PBMCs was analyzed by flow cytometry and (b) the data were statistically analyzed.* P < 0.05;* * P < 0.01;* * * P < 0.001.The experiment was repeated three times, and the data were expressed in the form of (mean ±SEM). Representative flow cytograms from 3 independent experiments were shown, and statistical histogram was quantified from 3 independent experiments.


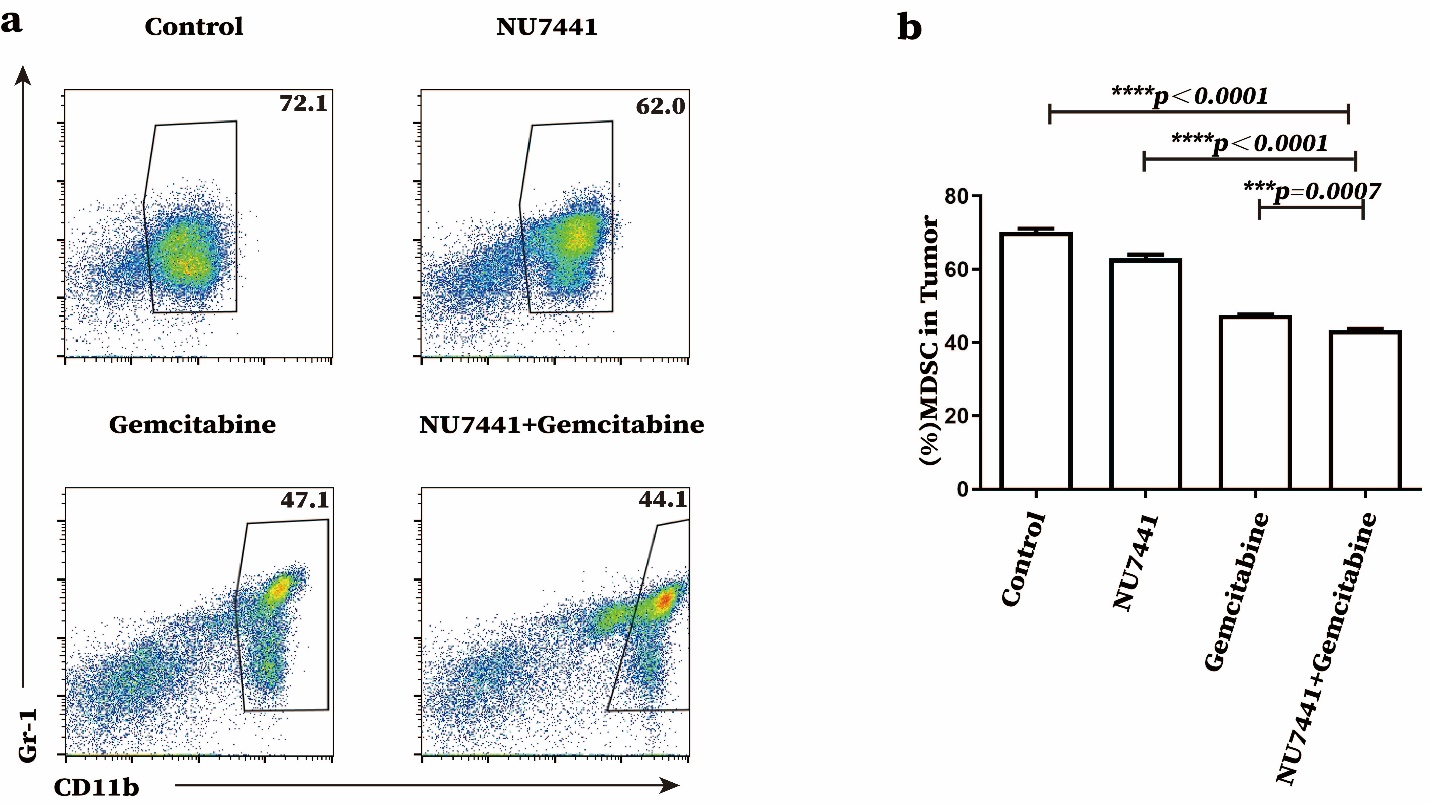


**Supplementary Figure 12. NU7441 combined with Gemcitabine significantly reduced** **the level of tumor infiltrating MDSCs.** After tumor-bearing, the mice were divided to 4 groups with 5 mice per group and intraperitoneally injected with DMSO or NU7441 at a dose of 10mg/kg for 5 consecutive days from day 8, intraperitoneally injected with Gemcitabine at a dose of 100mg/kg on day 3, day 6, day 9 and day 12. After NU7441 and Gemcitabine were given, the mice were sacrificed at day 20, and their tumors were removed to isolate tumor infiltrating immune cells. (a) The level of tumor infiltrating MDSCs was analyzed by flow cytometry and (b) the data were statistically analyzed (We tried our best to ensure the homogeneity of the MDSCs gate, but it was difficult to strictly guarantee the homogeneity of the MDSCs gate due to the heterogeneity of the tumor infiltrating MDSC in mice in different drug treatment groups, but we could still ensure the accurate differentiation of MDSCs cell population). * P < 0.05; * * P < 0.01; * * * P < 0.001. The experiment was repeated three times, and the data were expressed in the form of (mean ±SEM). Representative flow cytograms from 3 independent experiments were shown, and statistical histogram was quantified from 3 independent experiments.


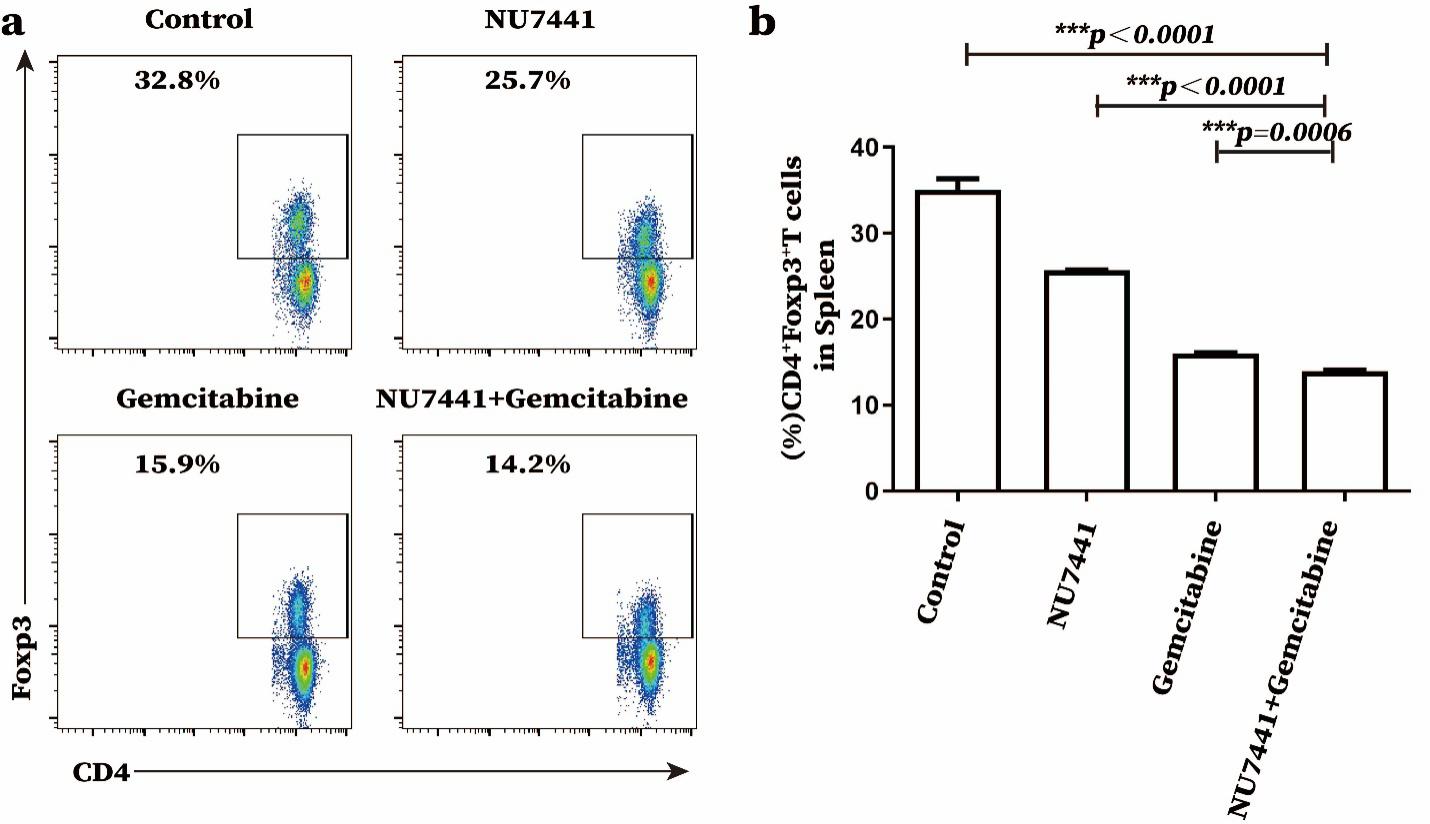


**Supplementary Figure 13. NU7441 combined with Gemcitabine significantly reduced the proportion of Treg in CD4^+^T cells in spleen of tumor-bearing mice.** After tumor-bearing, the mice were divided to 4 groups with 5 mice per group and intraperitoneally injected with DMSO or NU7441 at a dose of 10mg/kg for 5 consecutive days from day 8, intraperitoneally injected with Gemcitabine at a dose of 100mg/kg on day 3, day 6, day 9 and day 12. After NU7441 and Gemcitabine were given, the mice were sacrificed at day 20, and their spleens were removed to prepare single-cell suspension. (a) The proportion of Treg in CD4^+^T cells in spleen of tumor-bearing mice was analyzed by flow cytometry and (b) the data were statistically analyzed. * P < 0.05; * * P < 0.01; * * * P < 0.001. The experiment was repeated three times, and the data were expressed in the form of (mean ±SEM). Representative flow cytograms from 3 independent experiments were shown, and statistical histogram was quantified from 3 independent experiments.


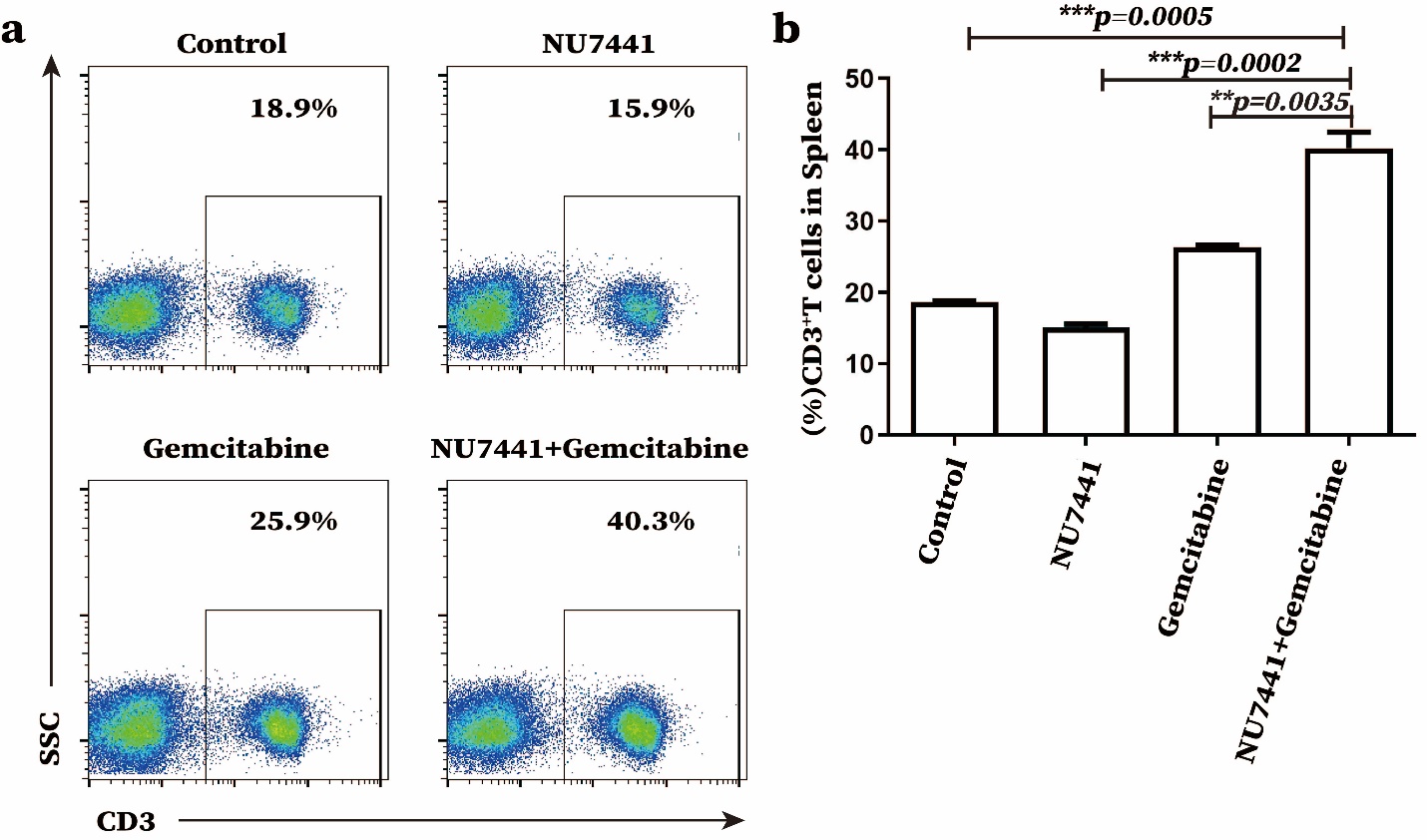


**Supplementary Figure 14. NU7441 combined with Gemcitabine significantly increased the proportion of CD3^+^T cells in splenic lymphocytes from tumor-bearing mice.** After tumor-bearing, the mice were were divided to 4 groups with 5 mice per group and intraperitoneally injected with DMSO or NU7441 at a dose of 10mg/kg for 5 consecutive days from day 8, intraperitoneally injected with Gemcitabine at a dose of 100mg/kg on day 3, day 6, day 9 and day 12. After NU7441 and Gemcitabine were given, the mice were sacrificed at day 20, and their spleens were removed to prepare single-cell suspension. (a) Flow cytometry was used to analyze the proportion of CD3^+^T cells in splenic lymphocytes from tumor-bearing mice and (b) to conduct data statistics. *P < 0.05; * * P < 0.01; * * * P < 0.001. The experiment was repeated three times, and the data were expressed in the form of (mean ±SEM). Representative flow cytograms from 3 independent experiments were shown, and statistical histogram was quantified from 3 independent experiments.


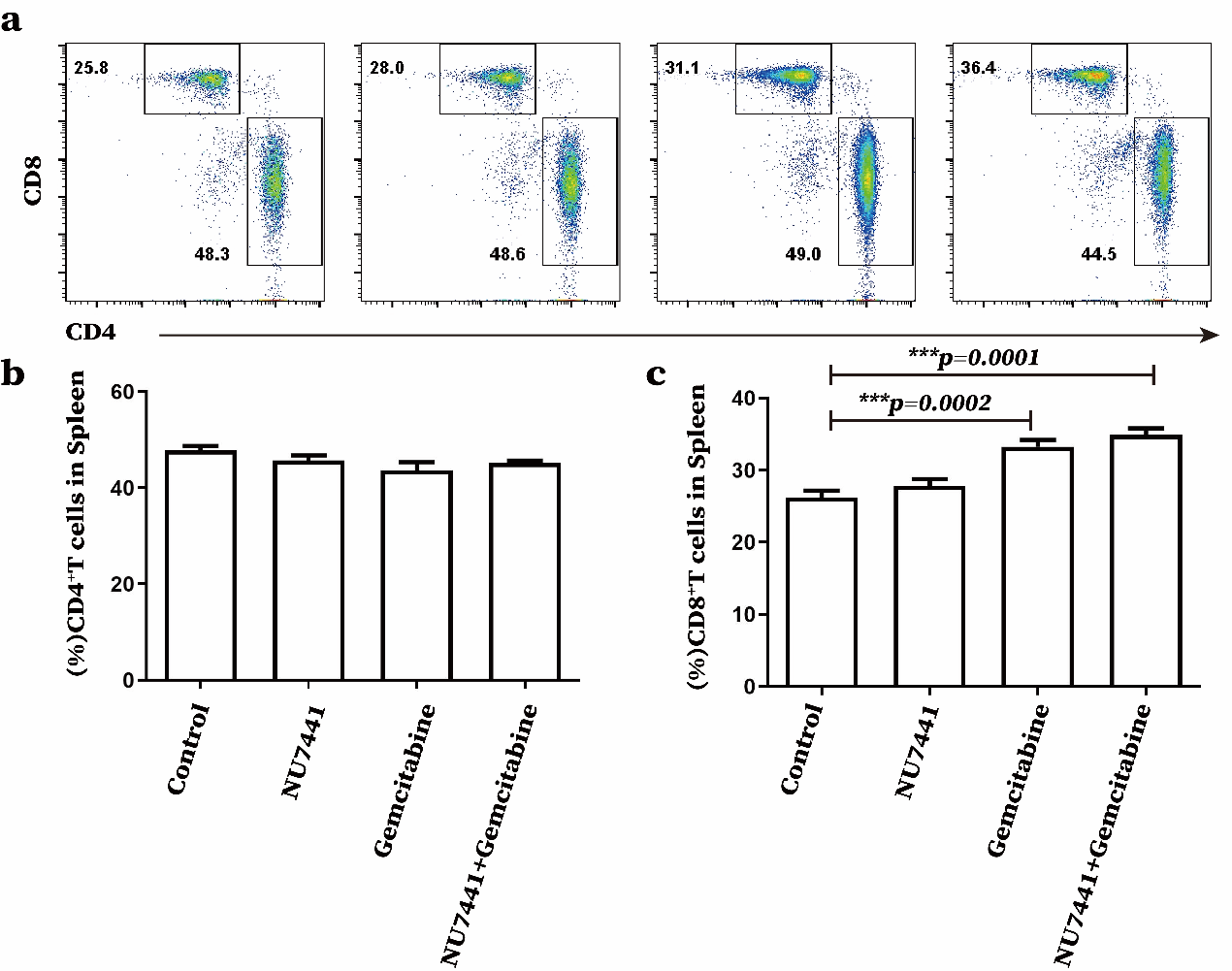


**Supplementary Figure 15. NU7441 combined with Gemcitabine did not affect the proportion of CD4^+^ and CD8^+^T cells in CD3^+^T cells in spleen of tumor-bearing mice.** After tumor-bearing, the mice were divided to 4 groups with 5 mice per group and intraperitoneally injected with DMSO or NU7441 at a dose of 10mg/kg for 5 consecutive days from day 8, intraperitoneally injected with Gemcitabine at a dose of 100mg/kg on day 3, day 6, day 9 and day 12. After NU7441 and Gemcitabine were given, the mice were sacrificed at day 20, and their spleens were removed to prepare single-cell suspension.(a) The proportion of CD4^+^ and CD8^+^T cells in CD3^+^T cells in spleen of tumor-bearing mice were analyzed by flow cytometry and the proportion of（b）CD4^+^ and （c）CD8^+^T cells in CD3^+^T cells in spleen of tumor-bearing mice were plotted.* P < 0.05;* * P < 0.01;* * * P < 0.001.The experiment was repeated three times, and the data were expressed in the form of (mean ±SEM). Representative flow cytograms from 3 independent experiments were shown, and statistical histograms were quantified from 3 independent experiments.
